# IMProv: A Resource for Cross-link-Driven Structure Modeling that Accommodates Protein Dynamics

**DOI:** 10.1016/j.mcpro.2021.100139

**Published:** 2021-08-19

**Authors:** Daniel S. Ziemianowicz, Daniel Saltzberg, Troy Pells, D. Alex Crowder, Christoph Schräder, Morgan Hepburn, Andrej Sali, David C. Schriemer

**Affiliations:** 1 Department of Biochemistry and Molecular Biology, University of Calgary, Calgary, Alberta, Canada; 2 Robson DNA Science Centre, Arnie Charbonneau Cancer Institute, University of Calgary, Calgary, Alberta, Canada; 3 Department of Bioengineering and Therapeutic Sciences, Department of Pharmaceutical Sciences, and California Institute for Quantitative Biomedical Sciences, University of California, San Francisco, California, USA; 4 Department of Chemistry, University of Calgary, Calgary, Alberta, Canada

**Keywords:** structural mass spectrometry, cryo-electron microscopy, crosslinking, hydrogen-deuterium exchange, integrative modeling, Polycomb Repressive Complex 2, Cryo-EM, cryoelectron microscopy, FDR, false discovery rate, HPC, high-performance computing, HX-MS, hydrogen exchange mass spectrometry, IMP, integrative modeling platform, PMI, Python modeling interface, PRC2, Polycomb repressive complex 2, SLURM, simple Linux utility for resource management, XL-MS, cross-linking mass spectrometry

## Abstract

Proteomics methodology has expanded to include protein structural analysis, primarily through cross-linking mass spectrometry (XL-MS) and hydrogen–deuterium exchange mass spectrometry (HX-MS). However, while the structural proteomics community has effective tools for primary data analysis, there is a need for structure modeling pipelines that are accessible to the proteomics specialist. Integrative structural biology requires the aggregation of multiple distinct types of data to generate models that satisfy all inputs. Here, we describe IMProv, an app in the Mass Spec Studio that combines XL-MS data with other structural data, such as cryo-EM densities and crystallographic structures, for integrative structure modeling on high-performance computing platforms. The resource provides an easily deployed bundle that includes the open-source *Integrative Modeling Platform* program (IMP) and its dependencies. IMProv also provides functionality to adjust cross-link distance restraints according to the underlying dynamics of cross-linked sites, as characterized by HX-MS. A dynamics-driven conditioning of restraint values can improve structure modeling precision, as illustrated by an integrative structure of the five-membered Polycomb Repressive Complex 2. IMProv is extensible to additional types of data.

## Highlights

• A resource for streamlining integrative structural modeling using XL-MS.
• A method for contextualizing crosslink restraints with HX-MS data.
• Improved model precision arises from dynamics-controlled cross-link restraints.
• A complete structural model of the PRC2 complex.

## Graphical Abstract

**Figure:**
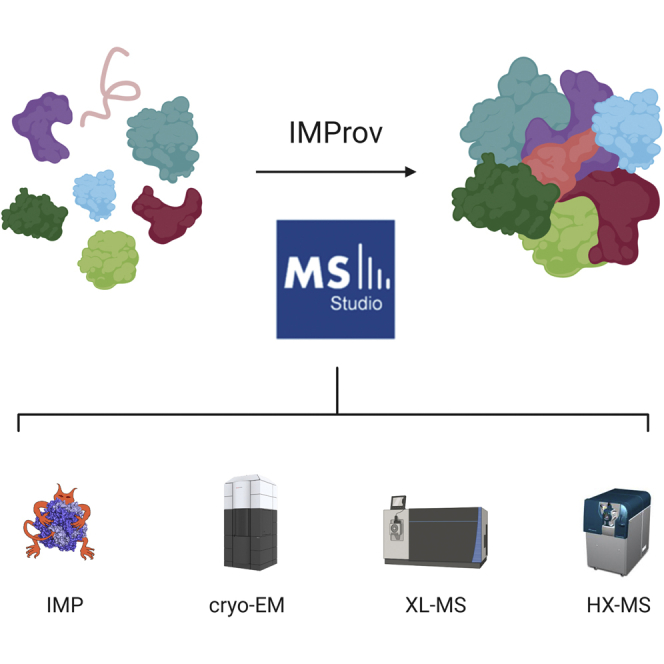

## In Brief

Proteomics has much to contribute to the structural analysis of cells, particularly considering the new developments in computational structural prediction. Cross-linking MS data can use structural building blocks (from empirical or modeled sources) to assemble models of higher-order complexes involving many proteins. In this work, we present a resource (IMProv) that reduces the barrier to integrative structural modeling and improves the precision of modeling exercises by incorporating dynamics (in the form of hydrogen/deuterium exchange data) to condition the cross-linking restraints.

Cross-linking mass spectrometry (XL-MS) is a popular technique for validating direct interactions in protein complexes, one that finds use in interactome analysis and in the structural characterization of large multiprotein complexes. XL-MS employs bifunctional reagents to covalently couple two residues within a distance permitted by the linker. Classically, the technique was used simply to stabilize complexes for the identification of new factors but with improved MS instrumentation and software, linked residues can now be detected with accuracy. This capability enables a deeper topological analysis of protein networks (1, 2, 3) and produces rich restraint sets for protein structure determination (4, 5, 6). The integration of cross-linking data with complementary structural techniques has the potential to generate accurate multiscale models of higher-order molecular processes. What XL-MS may currently lack in restraint precision is partially offset by leveraging existing structures and the scalability of the technique (7). For example, X-ray crystallography can reveal the atomic resolution of most individual protein substructures, but XL distance restraints can support the assembly of potentially hundreds of substructures into an accurate model of entire functional organizations (8, 9, 10). Even the accuracy of standalone biophysical techniques can be improved with XL data. Studies have shown that cross-linking data can help overcome positional ambiguity in modest-resolution cryo-EM density maps and SAXS envelopes (9, 11, 12, 13).

Given its potential, proteomics labs have significant opportunities to drive structure determination projects. A wide variety of resources are available, including improved cross-linking chemistries (1, 14, 15, 16, 17, 18, 19), sample processing routines (2, 20, 21, 22), quantitative methods (23, 24), and tools for data processing and analysis (25). Some structure modeling packages can accept cross-linking data (26, 27, 28), but there are few resources that seamlessly interface with the raw data generated by XL-MS and at the same time accommodate the complexities of such experiments. It is not at all obvious how best to use cross-linking data in modeling. Cross-links are typically modeled as static distance restraints that inform a pseudo-energy function (29, 30), the minimization of which reduces the distance between residues within a protein system. The maximum distance between linkage sites is coarsely modeled as a maximum Euclidian distance cutoff value, theoretically determined based on cross-linker chemistry. More nuanced approaches take into account residue side chain dynamics and solvent accessibility (29, 31) or tolerate conflicting restraints in order to identify alternative conformations (26, 32).

However, there are problems with current approaches to modeling. Flexible and dynamic protein systems sampled with the usual long-lived reaction chemistries are susceptible to kinetic trapping, a phenomenon where conformational transitions are cross-linked, resulting in restraint sets that are not representative of the equilibrium structure (33, 34, 35). Such heterogeneous structural data result in models with poor accuracy and precision and may preclude model convergence entirely (35). Our current inability to accurately account for the slow rate of cross-link accrual likely explains why many applications of XL-MS simply involve a rudimentary *evaluation* of models generated by other methods, rather than driving modeling itself (36, 37, 38). When measured cross-link distances exceed expectations, they are characterized as “overlength” or “violations.” These cross-links are typically reasoned away by postulating structural dynamics or dismissed as false-positive assignments. To improve outcomes, new methods are needed to detect these violations prior to modeling.

In this work, we provide a new resource for configuring modeling exercises directly from a mass spectrometry data analysis platform and suggest a new strategy to address the effect of underlying protein dynamics on cross-linking. We propose that direct measurements of local protein flexibility, using hydrogen–deuterium exchange MS (HX-MS) for example, could allow for the development of a dynamic restraint function. HX-MS measures the stability of hydrogen bonding networks—a major energetic component of structural stability (39, 40, 41)—by monitoring the hydrogen exchange rates between backbone amides and D_2_O in bulk solvent (42, 43).

HX-MS is not straightforward to use in modeling based on the complexity of the exchange event. As solvent accessibility is not the primary influencer of exchange rates, it must be used with caution in modeling protein interactions, as binding events can also induce distal changes in protein stability (43, 44). However, it excels at detecting stability at high resolution and has been used to map and model secondary structure and protein folding (45, 46). These capabilities stimulated us to evaluate its utility in determining the underlying structural stability of cross-linked sites. HX-MS analysis of large protein systems is now possible and data can be collected alongside of XL-MS and cryo-EM experiments (47, 48). We describe a new and freely available app available in the Mass Spec Studio (IMProv, www.msstudio.ca) that configures a modeling run for IMP (www.integrativemodeling.org) (26). The app supports the integration of cryo-EM densities, existing structures and cross-linking data, with the option of generating distance restraints conditioned by HX-MS. The functionality of the app is described and tested by modeling the five-member Polycomb Repressive Complex 2 (PRC2) (49, 50, 51), a 300 kDa chromatin remodeling complex.

## Experimental Procedures

### Protein Preparation

The preparation of PRC2 was as described in a previous study (52). Briefly, DNA fragments encoding human full-length EED, EZH2, RBAP48, SUZ12, and AEBP2_209–503_ were cloned into pMFSIC-T (a modified version of the pFastbac l vector) as TEV cleavable C-terminal fusions with maltose-binding protein. Viruses generated from the five individual constructs were used to coinfect sf21 cells at a 0.4:1:0.4:3:0.6 ratio for 72 h. EZH2 five complex was purified from frozen cells with maltose resin in a buffer containing 25 mM Tris, pH 7.4, 250 mM NaCl, 1 mM TCEP, and Roche EDTA-free protease inhibitor cocktail. Maltose-eluted protein complex was then treated with TEV protease and lambda phosphatase overnight. Nontagged and nonphosphorylated EZH2 complex was further purified by ion exchange on a HiTrap Q column and then by size-exclusion chromatography on a Sephacryl S300 column in 10 mM Tris, pH 7.4, 250 mM NaCl, 2 mM TCEP. The complex was concentrated to 19 mg/ml before flash freezing small aliquots and storage at −80 °C.

### HX-MS

Stock solution of PRC2 was diluted to 5 μM in 10 mM Tris-HCl (pH 7.4), 250 mM NaCl and 2 mM TCEP. For equilibration, the complex was incubated for 1 h on ice prior to performing HX-MS analyses. Experiments were carried out by adding an equal volume of 90% D_2_O (in 10 mM Tris-HCl (pH 7.4), 250 mM NaCl and 2 mM TCEP) to 2.5 μl of 5 μM protein solution at 4 °C. Three different deuteration time points for labeling (1 min, 10 min, and 100 min) were acquired in three independent replicates. For protein digestion under quenching conditions, 1 μl sample was added to 4 μl of 10× concentrated pitcher fluid from Nepenthes sp, dissolved in 100 mM Gly-HCl, pH 2.5 (53). Digestion was performed at 10 °C for 2 min. Proteolytic digests were directly loaded onto a self-made preconcentration column at 4 °C, followed by a 10 min gradient of 10 to 40% acidified acetonitrile, using an Eksigent nanoLC-ultra-2D pump coupled to a TripleTOF 5600 (Sciex) as described in detail elsewhere (47).

Database-driven peptide identification for library generation was performed with MASCOT v2.4 (Matrix Science). Briefly, fragment ion spectra were searched against a custom database containing all five PRC2 proteins with a precursor mass tolerance of 20 ppm and a fragment ion mass tolerance of 0.05 Da, with a probability cutoff of *p* = 0.05. Enzyme specificity was set to “none,” and no fixed or variable modifications were selected. The preliminary peptide library was imported into the Mass Spec Studio for further filtering and deuteration analysis (54). Spectral quality was assessed based on absolute intensity, signal-to-noise ratio, and spectral overlap with coeluting peptides on the MS1 level. Only those demonstrating complete isotopic envelopes were kept. Relative deuteration levels were manually validated and exported from the Studio. Calculation of protection factors was performed as described elsewhere (47). HX-MS measurements were collected in triplicate at all three time points.

### XL-MS

For chemical cross-linking, PRC2 was buffer exchange into 10 mM HEPES pH 7.4 with all other components unchanged. Cross-linking was performed for 10 min at room temperature with DSS or BS^3^ at a 1:200 protein:reagent mass ratio at 2 mg/ml protein concentration. Final concentration of DMSO was 5.3% for DSS cross-linking; no DMSO was present for BS^3^. Cross-linking was quenched with 100 mM ammonium bicarbonate. Cross-linked protein was then denatured by the addition of 6 mM DTT and incubated for 30 min at 55 °C, followed by alkylation with the addition of 16 mM iodoacetamide for 30 min in the dark at room temperature. Trypsin was added at a 1:20 protein-to-enzyme mass ratio and incubated for 3.5 h at 37 °C. Tryptic digests were quenched with 0.5% formic acid (ACS reagent grade ≥98%, Thermo Scientific) to yield a final concentration of 1.66 μM.

One microliter of the quenched digest was loaded on an Acclaim PepMap 100 C18 guard column (75 μm × 2 cm, 3 μm particles, 100 Å; Thermo Scientific) by an nLC-1200 (Thermo Scientific) and separated by a PepMap reverse-phase C18 (75 μm × 50 cm, 2 μm particles, 100 Å; Thermo Scientific) at 55 °C. Peptides were eluted at 300 nl/min on a 60-min 5 to 40%B gradient. Mobile phase A consisted of 0.1% v/v formic acid in 3% acetonitrile (LC-MS grade; Thermo Scientific), mobile phase B consisted of 0.1% v/v formic acid in 80% acetonitrile. Data from an Orbitrap Fusion Lumos (Thermo Scientific) were acquired in OT/OT mode. Parameters were as follows: spray voltage was set to 2.0 kV and transfer capillary temperature set to 300 °C. MS scans were acquired at a 120,000 resolution and 350 to 1250 Th mass range. Cycle time was set to 3 s, where the most intense ions above an intensity of 5.0 × 10^4^ in charge states 4 to 8 were selected for fragmentation by HCD using a quadrupole isolation width of 1.5 Th and NCE = 32%. MS/MS data were acquired with a 15,000 resolution and a 1.0 × 10^5^ target AGC and 100 ms maximum injection time.

### XL-MS Data Analysis

Raw MS data was processed using CRIMP in the Mass Spec Studio (version 2.2) (55). XL-MS data analysis was as follows: cross-link insertion was limited to K and KSTY residues on the linked peptides (*i.e.*, only one non-K linkage allowed). XL–MS data was researched against a database of the five PRC2 proteins. Default parameters were used with the following exceptions: MinCharge = 3, MaxCharge = 8, ChargeStatesString = 1,2,3, PercentEValueThreshold = 50, MS MassTolerance = 5 ppm, MS/MS MassTolerance = 5 ppm, ElutionWidth = 0.33. Cross-linked peptide spectral matches were manually validated. Validated cross-links with an estimated FDR ≥0.5% were forwarded for modeling.

### IMProv

A new app was constructed in the Mass Spec Studio framework to manage the intake of modeling data (cryo-EM densities, atomic structures, XL-MS data and HX-MS data) and launch IMP modeling runs. The app contains a wizard to upload protein sequences, atomic structures, all available modeling data, and the necessary graphical interfaces for setting the protein topology files and the configuration of IMP. The output is a set of scripts generated from basic templates and configured by the user. These are combined with a container that includes IMP and the environment for running it. The template script for the IMP Python Modeling Interface (PMI v2) supports cryo-EM and XL–MS data as well as basic physical restraints, such as sequence connectivity and excluded volume. PMI also supports a number of other types of data and statistically derived spatial restraints, including SAXS, FRET, NMR, and pairwise statistical potentials (56).

IMProv provides an easy way to parameterize restraints and to configure the number of states to model, the number of frames sampled, and the number of replicas in Gibbs sampling. For example, multiple cross-linkers can be included simply by providing one data file per crosslinker and its associated default distance restraint. A topology file is also configured, which informs IMP model representation (*e.g.*, bead size and rigid body assignment). For each parameter, reasonable values are set by default to guide novice users *e.g.*, 10-residue bead size, 10,000 sampling steps, automatic rigid body assignment, etc. Finally, a SLURM script is generated to optionally execute and manage the replicate modelling runs on high-performance computational resources. The current SLURM script template can be adjusted according to the user’s resource allocation and modeling requirements. Local instances on a personal machine can be run directly by executing the python script in the appropriate local environment. The above scripts and topology file are outputted to a hierarchal data directory that includes the data files identified by the user and any resources fetched from the Protein Data Bank and/or EM Data Bank. The tutorials for using IMProv and to execute of the deployment bundle can be found on www.msstudio.ca and in the supplemental Data.

### Conditioning Cross-linking Restraints

We formulated two approaches to implement stability-conditioned distance restraints, termed *offset* and *slope*. The conventional cross-linking restraint scoring function in IMP applies a length parameter, based on the size of the crosslinking reagent, as the center of a sigmoid function that scores a cross-link distance violation (score penalty) (10). This length parameter is generally constant for each XL-MS cross-linking reagent. In the *offset* method we use the conventional restraint function but use a unique length value for each stability class. Specifically, the linkages between stable regions of structure are assigned a lower length threshold than the linkages between unstable regions of structure. In effect, the midpoint of the scoring function is scaled without affecting the shape of the function. The *slope* method differs in that it modulates the uncertainty in the position of cross-linked residues based on a stability measure. In the conventional scoring function, structural uncertainty in each cross-linked residue is represented using a parameter, σ, which defines a spherical Gaussian around the center of the residue coordinate. For the slope method, regions of high structural uncertainty are assigned large σ values, resulting in a flattened scoring function that is more tolerant of overlength cross-links. Using a measure of local stability, such as one based on HDX data, a prior distribution can be assigned to σ that incorporates this information. Because it is unclear how structural stability measurements should be translated to physical distance restraint modifications, we formulated a function to sample the value of *σ* according to the Gamma distribution, which follows the form:

$$f\left(\sigma ;\kappa ,\theta \right)=\frac{x^{\kappa -1}e^{-\frac{\sigma }{\theta }}}{\theta^{\kappa }\Gamma \left(\kappa \right)}$$

where Γ(κ) is the gamma function evaluated at parameters *κ* and *θ*. We use a constant scale parameter value of *θ* = 2, whereas the shape parameter, *κ*, varies with the HX stability class from 1.0 to 2.0. This allows for a data-driven setting of *σ* from a reasonable initial value.

The resources in the Mass Spec Studio for processing HX data and XL data were augmented with new analysis and export utilities to generate inputs that conform to the requirements of IMProv. Briefly, HX-DEAL (the app for generating and validating deuteration measurements) was upgraded to include a Bayesian HDX tool for aggregating all overlapping sequences and returning per-residue protection factors (57). The tool was installed in the Mass Spec Studio *via* its embedded Python environment. We used the pre-merged 2.1 branch available from github.com/salilab/bayesian_hdx/tree/v2.1. In CRIMP (the app for identifying and validating peptide crosslinks), we developed an aggregator to collapse site identification redundancy and accommodate ambiguous site identifications. Briefly, cross-link spectrum matches (CSMs) were grouped across features and filtered according to a user-defined separation value (delta score) to address conflicts with other peptide types (*e.g.*, dead ends). A conflicting match within the delta value leads to the removal of CSM. CSMs with conflicts at the site level were reduced by score filtering with a user-defined separation value (gamma score) to advance a single linked residue pair. Unique CSMs were identified for all runs separately and combined into a single list.

### PRC2 Modeling from IMProv

Integrative modeling of the pentameric PRC2 complex was performed using the XL-MS and HDX-MS data described above, along with additional experimental information. An EM map of the PRC2 complex (EMD: 2236, resolution of 21 Å) was used to define the overall shape of the complex. Two recent atomic structures of PRC2 (PDB: 6C23, 5WAI) were combined and used for benchmarking and validation. The positions of residues in individual subunits were informed by crystallographic atomic structures of the truncated EZH2-SUZ12-EED complex (PDB: 5HYN). A comparative protein structure model of AEBP2 was computed with Modeller (58), based on an alignment from HHpred Server from the MPI Bioinformatics Toolkit (59). A comparative model of RBAP48 was constructed using Modeller, based on a homolog of RBAP48 from *Drosophila* with 90.6% identity (PDB: 2YB8).

The structure of PRC2 is represented as spherical beads of various sizes. Residues described with high-resolution input data were represented simultaneously as beads corresponding to one and ten residues. The balance of the model was represented solely as beads of up to ten residues. Components are also represented by 3D Gaussians to fit against the EM map (60). Beads and Gaussians were either arranged as flexible strings, based on sequence connectivity, or in a rigid body as defined in atomic models. A rigid body fixes the relative distances between the beads and Gaussians in the domain. The PRC2 complex contained three rigid bodies, including EZH2-SUZ12-EED, AEBP4, and RBP4.

A scoring function was created to rank alternative configurations of the PRC2 components based on the input information. The scoring function contains four terms: 1) excluded volume restraints, 2) sequence connectivity restraints, 3) EM density restraint corresponding to the cross-correlation of model Gaussians to the EM map, and 4) chemical cross-linking restraints formulated as described above using HDX data to inform local structural variability. Structures of PRC2 were sampled using a Gibbs sampling method accelerated *via* Replica Exchange (61). Each frame was recorded after an interval of ten Monte Carlo steps. A total of 1.05 × 10^6^ frames were produced in 28 independent trajectories: 14 trajectories of 50,000 frames using 20 replicas and 14 trajectories of 25,000 frames using 40 replicas. Scoring functions were modified as described below, where we note that psi parameters were deactivated throughout.

Model ensembles generated above were filtered for fit to input information and assessed for sampling precision using previously described methods (62). Model validation was performed using the IMP-sampcon scripts available at https://github.com/salilab/IMP-sampcon. Clustering was performed with a grid size of 2 Å. The precisions of resulting clusters were characterized by the average RMSD to the centroid structure of the cluster. The accuracy of each cluster was defined as the averaged ensemble RMSD of the cluster to the high-resolution PRC2 benchmark.

### Preparation of the High-Resolution PRC2 Benchmark

A cryo-EM structure (PDB: 6C23) and a crystallographic structure (PDB: 5WAI) of PRC2 were combined to produce a hybrid structure with 67.5% sequence coverage (1789/2652 residues with coordinates). These two structures were combined *via* alignment of the RBAP48 subunit; N-terminal domain residues of SUZ12 in 6C23 were missing residue-resolution data, which were replaced with the residue-resolved data from the crystallographic structure 5WAI. Benchmarking against the high-resolution reference structure was performed by calculating the RMSD between the center of a bead from an IMP model to the centroid of the alpha-carbons of the residues represented by that bead in the reference structure. Accuracy could only be calculated for a subset of the PRC2 sequence in the integrative models because of missing residues in the high-accuracy structure.

## Results and Discussion

### IMProv, Structure Modeling from the Mass Spec Studio

**Fig. 1:**
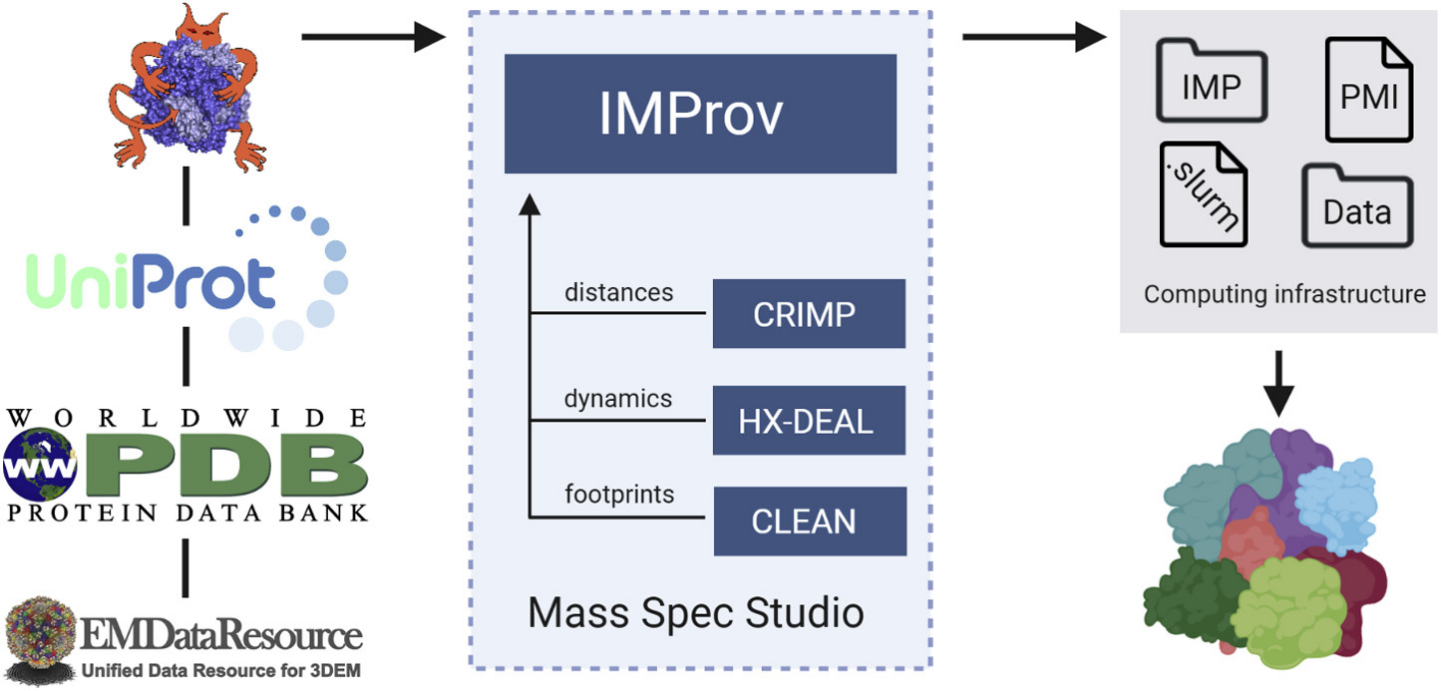
**IMProv, a workflow for integrative structural modeling in the Mass Spec Studio.** A graphical user interface enables the configuration of integrative modeling routines based on IMP (26). IMProv works in concert with MS data analysis apps in the Studio to receive XL and HX datasets that are properly configured for use within IMP, with an option for footprinting data as well. These datasets are combined with structures from the Protein DataBank (PDB), available cryo-EM density maps (EMD), and sequence information as needed. A deployment bundle including IMP and its dependencies is exported, which can be executed on a high-performance computing cluster for integrative modeling of the protein system.

To initiate protein structure modeling exercises with structural mass spectrometry data, we created IMProv in the Mass Spec Studio (63). The Studio contains apps for both XL-MS (CRIMP) and HX-MS (HX-PIPE and HX-DEAL), which were used to process data for the modeling runs described below. The new app supports integrative modeling with a familiar wizard-style user interface that helps the user set up and configure an IMP-based modelling exercise (Fig. 1). Briefly, the user provides sequence information for each protein “building block” and any available structure files (partial or homologous). These are represented in modeling according to the resolution of the individual structural unit. The user can specify any level that is appropriate, from fully structured to completely ambiguous, and on a domain-by-domain basis. Data sets from multiple restraint generators can then be associated with the modeling run. For XL-MS data, multiple cross-linkers and default distance restraints can be configured. In our lab, a typical and well-tested modeling run combines XL-MS data and electron microscopy densities.

IMProv outputs four components necessary for modeling. First, it generates a script for the IMP Python Modeling Interface (PMI), customized according to the user input. Second, it creates the corresponding data directories. Third, it produces an SLURM bash script to execute modeling on a high-performance computing (HPC) cluster. Convergent sampling of large models within a practical time frame often requires access to HPC infrastructure. Therefore, in the fourth component, the script is combined with a deployment bundle that contains IMP and the requisite software in a correctly configured environment, based on the SLURM workload manager. This will be of particular value to novice users. The online tutorials provide the details required for effective deployment. Because computational resources and technical support are not always readily available to users, we support a maintained installation on communal Compute Canada resources and have included a configured bundle for deployment on Amazon Web Services (www.msstudio.ca). All that is required is the requisite input data for IMProv and basic skills in Linux to execute the deployment bundle and perform integrative modelling. Advanced users of IMP can alter the PMI script with custom or optimized functions (www.integrativemodeling.org).

### PRC2—A Test Case for IMProv

IMProv was then used as a platform to explore new ways of integrating mass spectrometry data for modeling. Cross-links formed between structurally dynamic regions can produce distance measurements that appear to exceed the natural constraint of the linker. We reasoned that some (if not all) of these motions may be detectable by HX-MS. To test this hypothesis, we collected XL-MS and HX-MS data on the Polycomb Repressive Complex 2 (PRC2). The complex consists of four structural subunits (RBAP48, EED, SUZ12, and AEBP2) that scaffold and activate EZH2. EZH2 is a histone H3 methyltransferase that regulates transcriptional repression of target genes (49, 64). PRC2 is a useful candidate for several reasons. First, PRC2 possesses regions of high stability as well as intrinsic disorder; the range of stability necessary to test our hypothesis. Second, data that are complementary to XL-MS are publicly available: a low-resolution cryo-EM map (65) and high-resolution crystallographic structures of some individual components (66, 67). Third, the performance of our strategy can be benchmarked against a recent high-resolution hybrid structural model generated by cryo-EM and X-ray crystallography (36, 68). However, approximately one-third of the PRC2 sequence is not represented in this structure, so there is an opportunity for an integrative approach to generate a complete model and new insights.

**Fig. 2:**
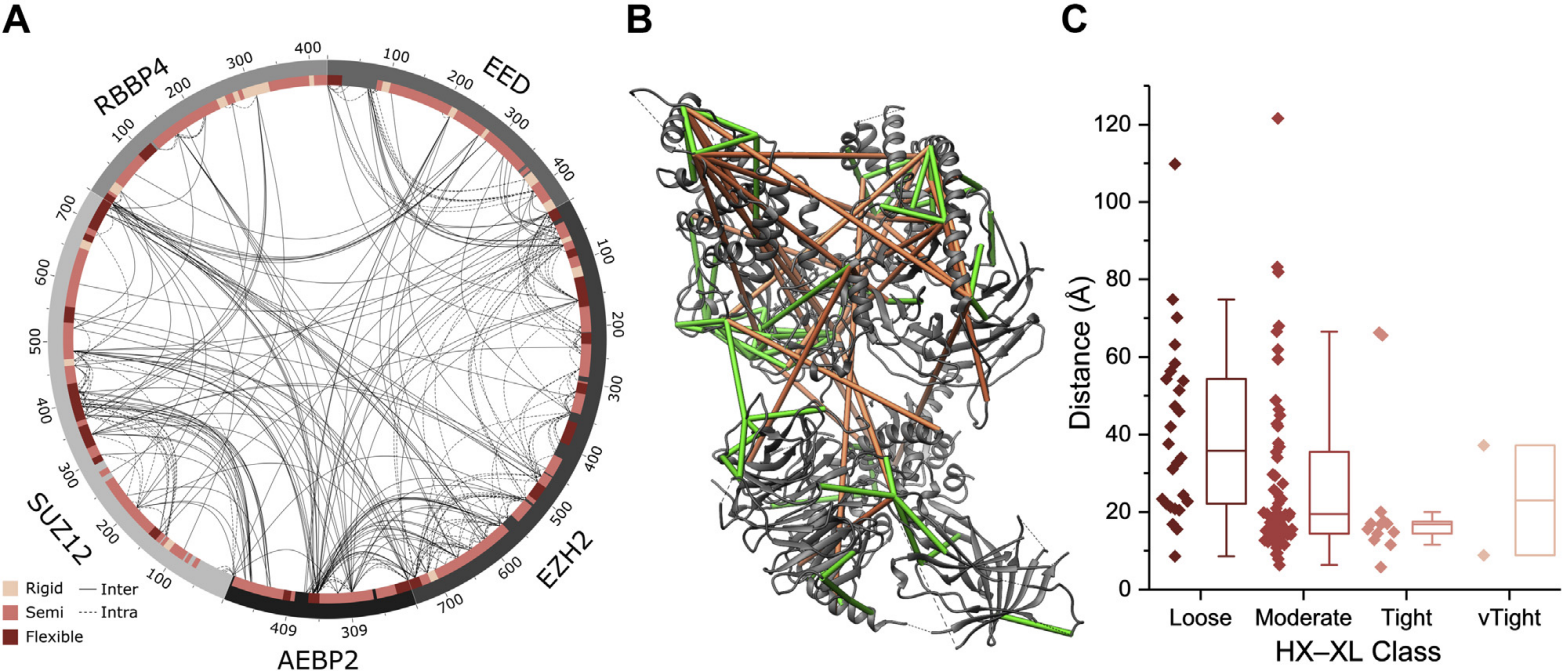
**Mapping XL-MS and HX-MS data onto PRC2.***A*, circos plot showing the aggregate of unique DSS and BS^3^ cross-linking sites, with stability zones marked on the inner ring according to the HX-MS data. *B*, unique cross-links displayed on the hybrid high-resolution PRC2 structure; *green* linkages are ≤35 Å, *orange* are ≥35 Å. Only cross-links that correspond to resolved structure are shown (approximately one-third of total XLs). *C*, box plot of cross-link distances grouped by stability class, as shown in (*A*). Bars indicate quartiles, whiskers indicate 1.5 × IQR (interquartile range).

**Fig. 3:**
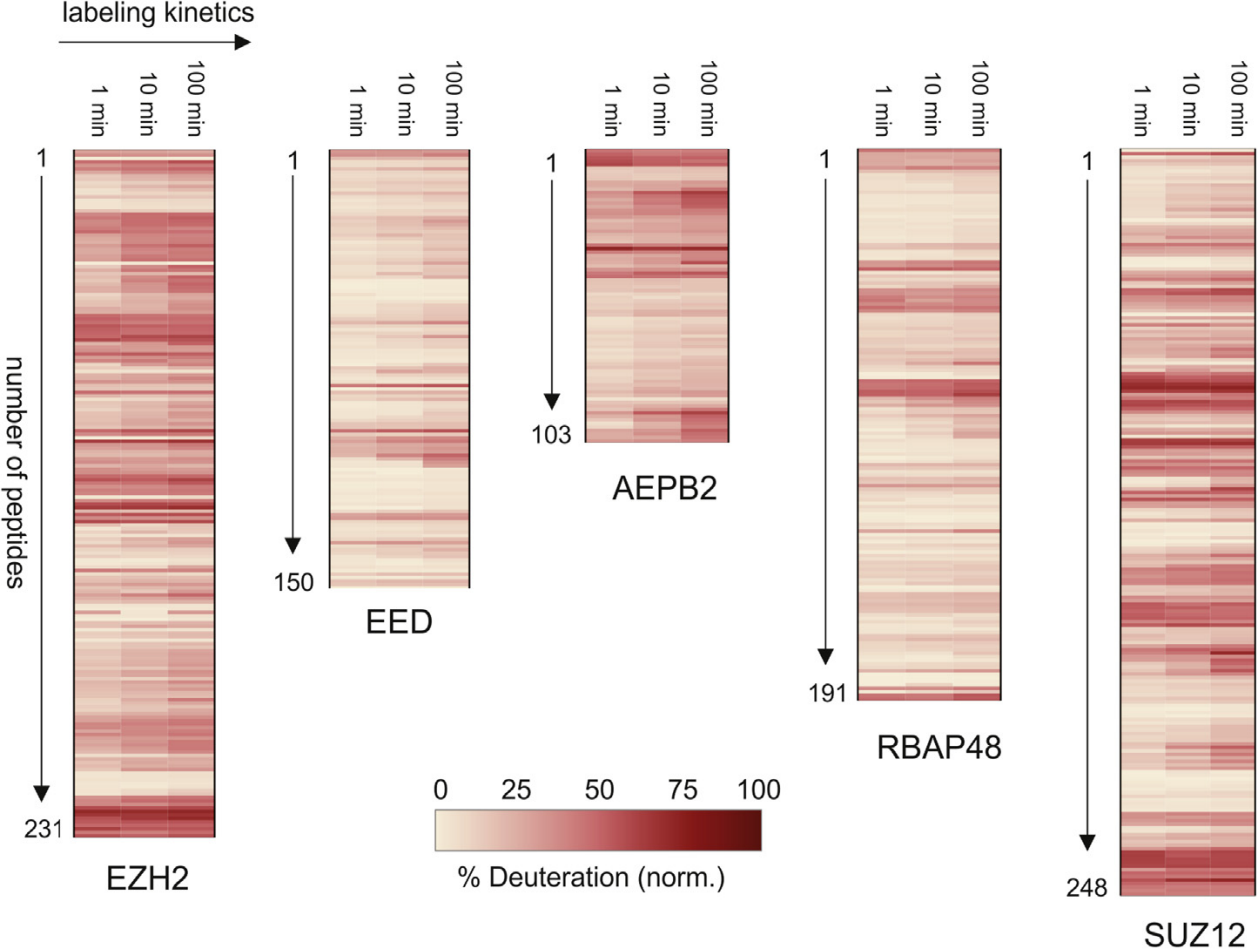
**HX-MS heat maps.** Data mapped onto the sequences of all five core PRC2 proteins, displaying 90% sequence coverage.

Cross-linking of PRC2 with DSS and BS^3^ produced 281 and 144 nonredundant cross-links, respectively, at an estimated FDR of 0.5% (Fig. 2*A*). Most of these cross-links are between lysines; only 5% are to serine, threonine, and tyrosine. AEBP2 was the most densely cross-linked subunit with a ratio of one cross-link per three residues (1:3), followed by EZH2 (1:4) and SUZ12 (1:6). RBAP48 and EED were the least cross-linked, with ratios of 1:11 and 1:9, respectively. These were calculated using all inter and intraprotein crosslinks. HX-MS datasets were collected on the complex as well, sampled at three time points to support a coarse dynamics assessment and the calculation of local protection factors (69). A total of 924 peptides showed measurable deuteration values, corresponding to an average sequence coverage of 90% and a range from 85% to 99% across all five proteins (Fig. 3). The kinetics data for each peptide were transformed into higher-resolution Protection Factors (PFs) using a Bayesian approach to aggregate overlapping sequences (57). The resulting assignments were manually collapsed into 101 discrete segments based on obvious stability boundaries (median length 16 residues, range 1–137 residues) and allocated into three categories: stable, semistable, and flexible (Fig. 2*A*), based on the natural scaling provided by protection factors. According to this approach, 61% of PRC2 sequence was graded as semistable, with 9% graded as stable, and 20% as flexible; measurements could not be obtained for the remaining 10%.

We then classified each unique cross-linking pair according to the “stability zones” that they spanned. The high HX-MS sequence coverage allowed us to sort all 336 unique crosslinks (*i.e.*, the intersect of DSS and BS^3^ linked sites) into one of five different classes: 2 very tight (between two stable regions), 24 tight (one stable and one semistable region), 120 moderate (between two semistable regions or between a stable and flexible region), 148 loose (one semistable and one flexible region), and 42 very loose (between two flexible regions). The cross-links were then mapped to the latest high-resolution structure, a hybrid model constructed from ∼4.5 Å cryo-EM structure (PDB: 6C23), and a 2.9 Å crystallographic structure (PDB: 5WAI) (Fig. 2*B*). The model covers 67.5% of the total sequence, which permitted the mapping of 97 of the 336 crosslinks.

**Fig. 4:**
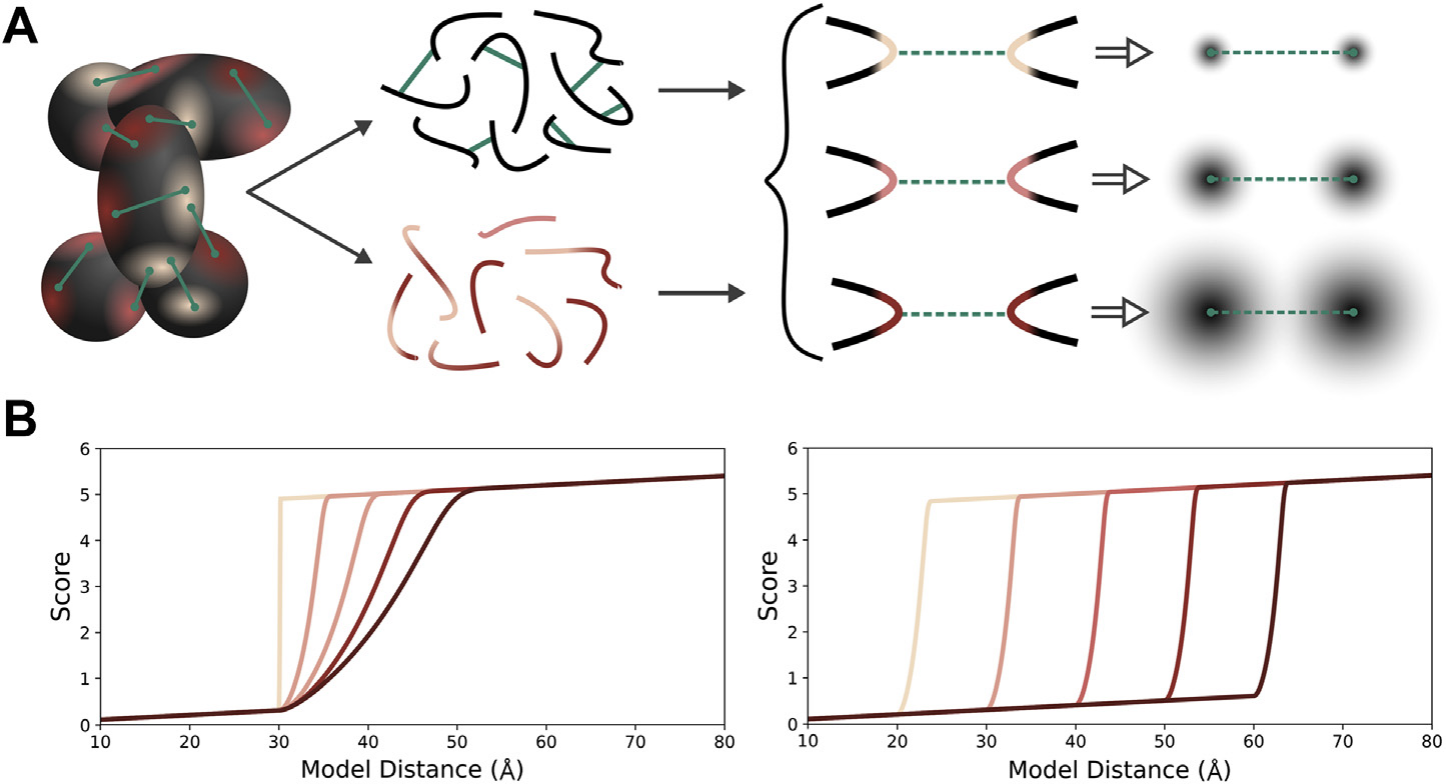
**Concept of conditioning of XL-MS distance restraints with HX-MS structural stability measurements.***A*, a protein complex is analyzed by both HX-MS and XL-MS methods. Both datasets are imported into the IMProv module of the Mass Spec Studio where cross-links are partitioned according to structural dynamics. A three-tiered example is shown. *Red*-*violet*-*blue* color scheme indicates stable, semistable, or unstable structural classes, respectively. Each class informs a scoring function based on a modified distance restraint, in this example presented as the ambiguity of a cross-linked site in space. *B*, altered distance restraint function based on the slope (*left*) and offset (*right*) methods. The slope method is related to the HX stability class

The classification of point-to-point distances according to stability supports the notion that HX may be able to predict the utility of the cross-linking restraint (Fig. 2*C*). To illustrate, the median measured cross-link distances for the three classes with sufficient data are: *loose* (35.8 Å), *moderate* (19.5 Å), and *tight* (16.8 Å). The delineation is not perfect. Outliers are observed in every category, likely reflecting the sampling of large, domain-level structural motions outside the scope of HX-MS measurements. Given the elongated and multilobed structure of PRC2, long-distance motions at the quaternary scale seem plausible. As expected, no cross-links could be mapped to structure from the large *very loose* class. These residues were not resolved in the high-resolution reference structure, consistent with their dynamic nature, and very likely reflective of low precision cross-linking distances. The median length and dispersion of the *tight* cross-links were well below the typical expected cross-linking distance for DSS/BS^3^ (30 ± 5 Å), suggesting that it may be useful to score such cross-links with a lower distance threshold. These observations support the idea that distance restraints could be set dynamically, according to the underlying structural dynamics as measured by HX-MS (Fig. 4*A*).

**Fig. 5:**
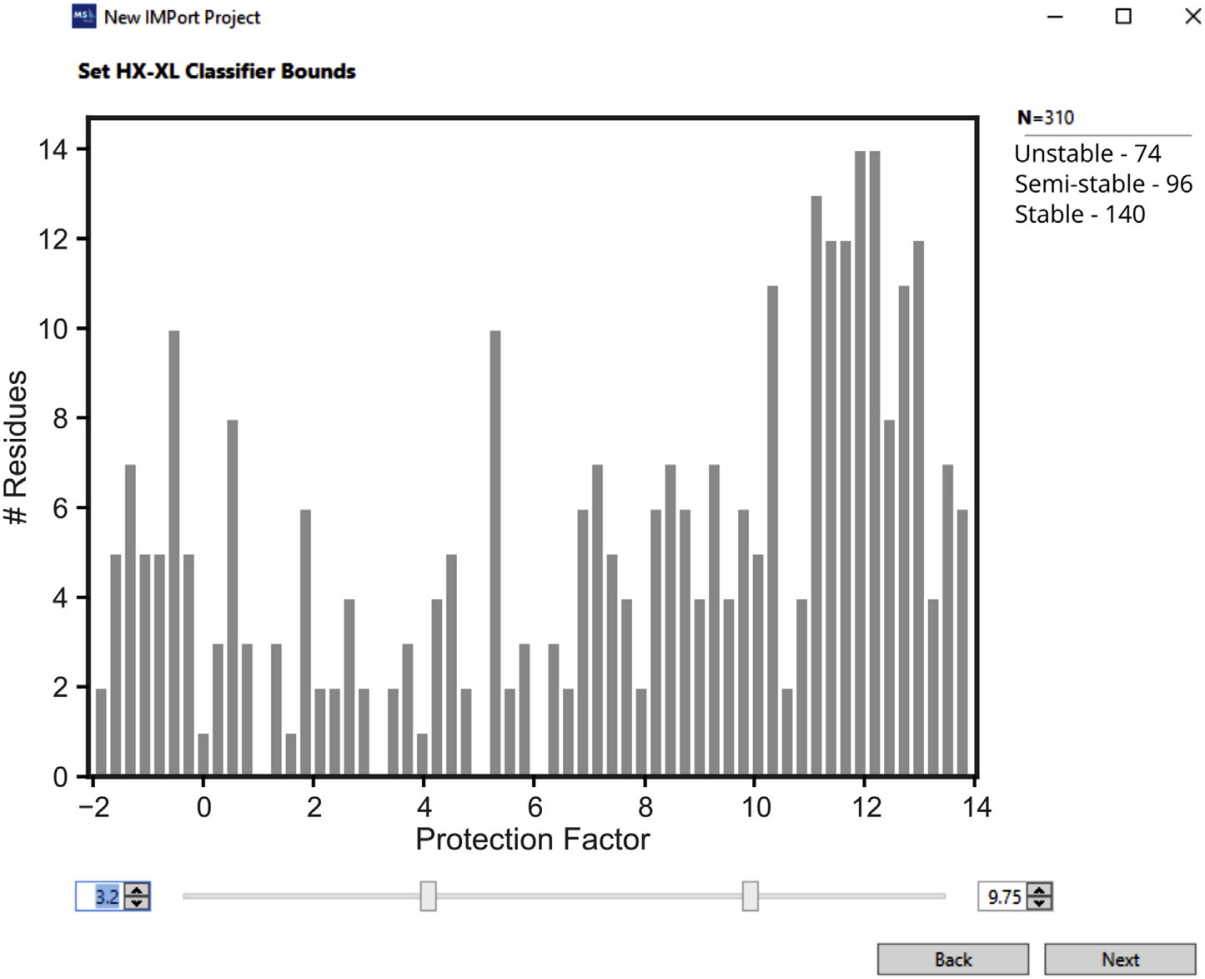
**Classification of cross-links with HX protection factors.** Screenshot of IMProv where the user selects protection factor cutoff values for downstream stability classification of cross-linked sites, guided by a graphical display of the composite protection factor values spanning the given sites.

To insert this capability into IMProv, we required two additional processing features: (1) an automated strategy to collapse redundant and overlapping HX data into contiguous segments with assigned stabilities and (2) a method to generate adaptive cross-link restraint functions that reflect the underlying local stability. For the first, as noted above, we calculated residue-resolved PFs from overlapping peptides using the Bayesian HDX algorithm designed by Saltzberg *et al.* (57). Although cross-linking provides residue-resolved linkages, it is not appropriate to condition cross-links with stability measures at the level of individual residues, because stability is a property of secondary and tertiary structure. To set zones of stability in an automated fashion, our approach involves Gaussian kernel smoothing of residue PFs, weighted according to the significance of the individual PFs and with a typical kernel size of five residues. In this way, linkage sites can be assigned more meaningful regional stability values. At the modeling configuration stage in IMProv, we then apply a coarse three-tier classification of stability (*i.e.*, stable, semistable, and flexible), based on user-defined thresholds (Fig. 5). Defaults were established with the PRC2 system. That is, the boundary between flexible and semistable was set to 3.2, creating a flexible set that roughly matches the population of missing structure in the cryo-EM models. The boundary between stable and semistable was set to 9.75 based on surveys of literature values for stable structure (*e.g.*, (70)). This creates the five classes of linkages that we presented above for PRC2 (very tight, tight, moderate, loose, and very loose). When HX data are not available for a region of sequence, a default tier of semistable is assigned.

We then tested a series of restraint sets using our two cross-link conditioning strategies and compared PRC2 modeling results with a control case, which used a conventional single restraint. Table 1 shows the parameterization of the full set of cross-linking restraint functions. The values were informed by the distribution of cross-link distances shown in Figure 2*B*. In the slope method, the σ values were restricted to a somewhat tighter range of distances than the offset method, because the restraint function in IMP demands that σ not exceed one-half of the “length” parameter. For modeling we chose to use the low-resolution density map of PRC2 (∼21 Å) over the recent high-resolution map (∼4.5 Å) for two reasons; first, to ensure that the relative weight of the cross-linking data would be maximized and second, to test if low-resolution cryo-EM structures could be accurately extended with cross-linking data.

**Table 1:** Values of the “length” and/or σ parameter values for HX-XL distance restraints

Restraint method	Method variant	Length/σ (Å)					
		Fixed	v.Tight	Tight	Mod.	Loose	v.Loose
Control	C1	30	-	-	-	-	-
	C2	40	-	-	-	-	-
	C3	50	-	-	-	-	-
Slope	S1	-	20/0.0	20/2.5	20/5.0	20/7.5	20/10.0
	S2	-	30/0.0	30/3.0	30/6.0	30/9.0	30/12.0
Offset	O1	-	25	30	35	40	45
	O2	-	20	25	30	35	40
	O3	-	20	25	30	45	60
	O4	-	20	30	40	50	60

Values of σ represent the maximum likelihood value of the Gamma distribution.

We first compared the results according to the sampling precision, as sampling precision ultimately dictates the precision of the modeling exercise, and the scale at which structural features can be inspected (71) (Table 2). The sampling precision of the control trials was quite poor, regardless of the cutoff used. For example, the conventional 30 Å value (C1) generated a precision of 49.6 Å with good clustering behavior. Progressively relaxing the global length restraint to 40 Å and 50 Å did not improve precision nor lead to any significant changes in clustering behavior. In most cases, the HX-conditioned restraints markedly improved the sampling precision and generated a well-populated major cluster. The O1 and O4 trials represent the best balance between high sampling precision and large cluster size. For example, O4 generated a sampling precision of 23.4 Å with a dominant cluster representing 86.9% of all models generated, much superior to the conventional 30 Å cutoff. Although the cluster precisions for the control trials are better than the associated sampling precisions, they cannot be used to define the precision of the modeling (even when the sampling precision is divided by √2 to support a direct comparison with cluster precision (71)). Thus, the HX-conditioned XL data generate significantly improved modeling results; for example, O4 with a cluster precision of 17.2 Å is much better than the 32.5 Å adjusted sampling precision of the C2 trial. These results suggest that selectively *increasing* the cross-link distance helps to avoid the generation of multiple clusters of solutions that can arise from overconstraining flexible regions. The results from the slope configurations are generally in keeping with the offset method but are somewhat less clear-cut. At this point, the slope method cannot be configured with large enough σ values to fully explore its utility.

**Table 2:** Clustering and statistical analysis of PRC2 integrative structural modeling results

Distance restraint	Trial	Clusters	XL satisfied^a^ (%)	Med. XL (Å)	Sampling precision (Å)	Cluster 1			Cluster 2		
						Size^b^ (%)	Cluster precision (Å)	Med RMSD^c^ (Å)	Size^b^ (%)	Cluster precision (Å)	Med RMSD^c^ (Å)
Control	C1	2	98	28.0	49.6	69.0	32.2	19.8	30.2	32.9	59.5
	C2	4	91	30.5	46.0	73.7	16.9	15.5	15.8	27.2	59.8
	C3	2	84	33.1	53.8	74.8	17.9	15.5	24.1	37.5	58.7
Slope	S1	4	97	26.9	30.2	90.4	24.5	32.8	6.1	23.8	32.4
	S2	9	98	27.8	22.2	63.6	16.8	36.2	24.5	17.4	37.8
Offset	O1	7	98	27.8	22.4	81.7	17	19.6	6.7	16.3	19.1
	O2	16	95	29.1	20.3	62.0	16.9	32.5	14.7	14.9	30.5
	O3	5	87	31.7	28.3	90.1	21.4	20.9	8.2	21.7	21.3
	O4	7	85	33.1	23.4	86.9	17.2	18.4	8.0	18.2	17.3

a Proportion of crosslink distance within 40 Å, between beads calculated from each RMF, from the cluster ensemble.

b Proportion of models clustered from the total of all clusters (30,000 total models clustered).

c Median RMSD value from the cluster ensemble.

**Fig. 6:**
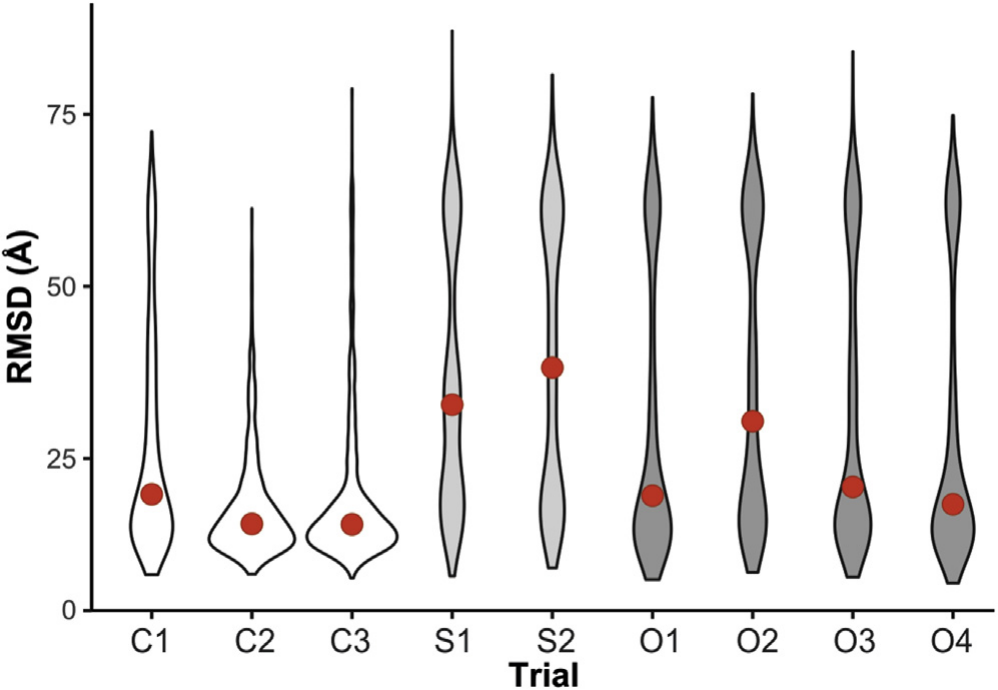
**Comparison of IMP models with benchmark structure.** Distribution of ensemble structure RMSDs for the main (*i.e.*, largest) cluster compared with the high-resolution benchmark structure of PRC2 (∼2/3rds of sequence). *Black bars* indicate medians.

We then compared results based on model accuracy using the high-resolution reference structure as a guide, recognizing that it represents only two-thirds of the entire protein complex. For the best offset configurations, modeling accuracy improved marginally compared with the conventional global crosslinking restraint of 30 Å (C1 configuration, Fig. 6), confirming that a dynamics-driven relaxation in cross-link precision can preserve modeling performance for the highly structured regions. We note that increasing the global cross-linking restraints to 40 Å and 50 Å slightly improved accuracy, but there is no *a priori* justification for modeling structures with distance restraint thresholds of 40 Å or higher. The slope method generated a solution set that was less accurate overall, again probably due to a restricted range of *σ* values. Taken together, although there is room for further exploration, stability-adjusted cross-link restraints can clearly generate accurate models while at the same time preventing an unjustified structuring of flexible regions.

### Structural Insights on PRC2

**Fig. 7:**
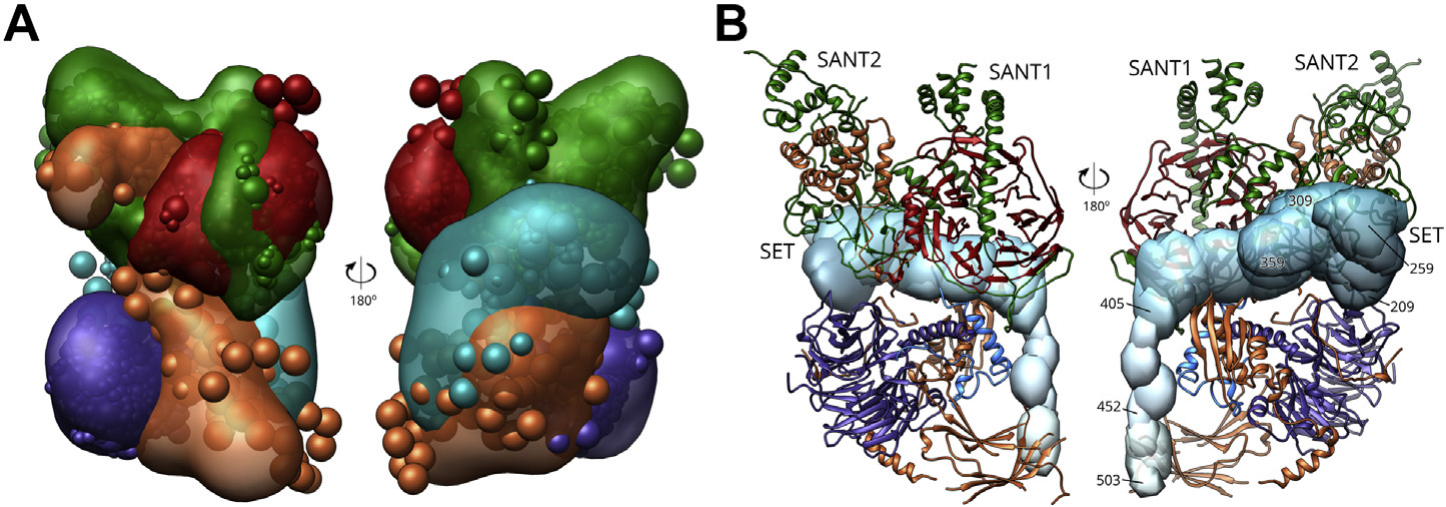
**Integrative modeling of PRC2.***A*, bead and density model of full-length PRC2, from the O-4 modelling trial. Subunits coloring: EZH2 in *green*, EED in *red*, RBAP48 in *violet*, SUZ12 in *orange*, AEBP2 in *dark cyan*. *B*, density of AEBP2_209–503_ from the O4 modeling trial overlaid on the high-resolution structure of PRC2 (PDB 6c23), colored as above, except with AEBP2_440–503_ in *blue*. Domain labels for EZH2 provided.

We chose to examine the model produced from the O4 trial, as it generates a clearly defined cluster of solutions, and because of its high accuracy with respect to the benchmark cryo-EM structure. The cross-linking data placed all structured elements at the correct locations, within the precision of the modeling exercise (Fig. 7*A*). In the benchmark, approximately one-third of the full-length PRC2 sequence is unresolved. Cross-linking localized much of the missing sequence, although several sequence gaps of 1 to 20 residues in length cannot be placed, because of the limits imposed by our cluster precision (17.2 Å, Table 2). The benchmark structure does not locate the majority of AEBP2. It was assembled using an AEBP2 isoform representing residues 209 to 503, but it could only position a small 64-residue region at the base of the EED subunit (AEBP2_440–503_). Our modeling maps out the rest of the subunit. Our complex was also constructed with AEBP2_209–503_. We demonstrate that it forms a more extensive binding surface, spanning the SET domain of EZH2 and tracking across EED to the “foot” of SUZ12 (Fig. 7, *A* and *B*). AEBP2 plays multiple roles in PRC2. It stabilizes the complex, mediates the folding of the SUZ12 zinc finger (36, 51, 65), and regulates PRC2 methyltransferase activity (72, 73). The extensive interaction around the waist of the complex is consistent with a stabilizing role, and the association with the SET domain is the likely means by which EZH2 activity is regulated (64, 65). Our model organizes a structured element (AEBP2_209–359_) proximal to the SET domain of the catalytic subunit, in keeping with a previous cross-linking study that places three zinc fingers in AEBP2 in this vicinity (65). The position of the extreme C-terminal tail is not in full agreement with the high-resolution structure (Fig. 7*B*), likely because of a lack of cross-links to the beta-sheet of SUZ12; however, the model density does place it within the correct structural quadrant.

In conclusion, IMProv provides a useful resource for combining cross-linking mass spectrometry data with other sources of data, to support structural modeling in a powerful integrative modeling framework. The resource makes this modeling capacity more accessible to proteomics laboratories that may not otherwise have experience in computational modeling. It also provides an opportunity for the more experienced structural biology labs to incorporate cross-linking data more effectively. Many applications of cross-linking are limited to simple validations of structures generated by other means (36, 37), because of the uncertainty surrounding mass spectrometric data interpretation. Distance restraints are usually estimated from simple and stable structures (29, 34, 74), but the *post hoc* mapping of XL-MS data on structures from a wider range of targets frequently shows subsets of overlength cross-links, making *de novo* modeling with cross-links a risky affair (4, 35, 38). We show that a data-driven assessment of structural dynamics can be used to adjust the restraints in a contextualized fashion: higher-precision restraints may be used for stable substructures and lower precision for less stable substructures. Additional test cases are needed to refine the approach, requiring more complexes for which both cross-linking and stability data are available. HX-MS offers ready access to local dynamics, but it is easy to see how this may be extended to other sources of stability analysis, whether computational or empirical. For example, larger domain motions are difficult to detect with HX-MS but could be determined through the use of both “fast” and “slow” cross-linkers (35). Together, such approaches can help avoid the current unfortunate practise of implementing a one-size-fits-all distance restraint, which both over and underestimates the value of the data.

Finally, this resource offers an effective tool for extending the value of existing and developing data repositories. Despite recent advances in EM hardware, many structures are still of moderate resolution with ill-defined regions, as the PRC2 example illustrates. Cross-linking data are generally easy to obtain and, when properly interpreted, provide an effective way to integrate archived structural data with any new structures that may emerge over time.

## Data Availability

All LC-MS and LC-MS/MS data generated to support the findings of this study (including all cross-links identified and their categorization, as well as tables supporting the HX-MS analysis) have been deposited to the ProteomeXchange Consortium *via* the PRIDE partner repository (75) with the dataset identifier PXD022861.

## Supplemental data

This article contains supplemental data.

## Conflict of interest

The authors declare that they have no conflicts of interest with the contents of this article.
