# Supplementary material for: IMProv: A Resource for Cross-link-Driven Structure Modeling that Accommodates Protein Dynamics
Source: Mol Cell Proteomics. 2021 Aug 19;20:100139. doi: 10.1016/j.mcpro.2021.100139 (PMC8452774; doi:10.1016/j.mcpro.2021.100139)
Supplement: Supplemental data [file mmc1.pdf]

IMProv project preparation

Presented by
MassSpecStudio Development Team

Introduction

- Overview of IMProv for IMP and PMI.
- Getting Started with the sample project (PRC2) on github.
- Prepare IMP Topology and Config files using MassSpecStudio.
- Deployment of the IMProv project on AWS, Cedar or PC.

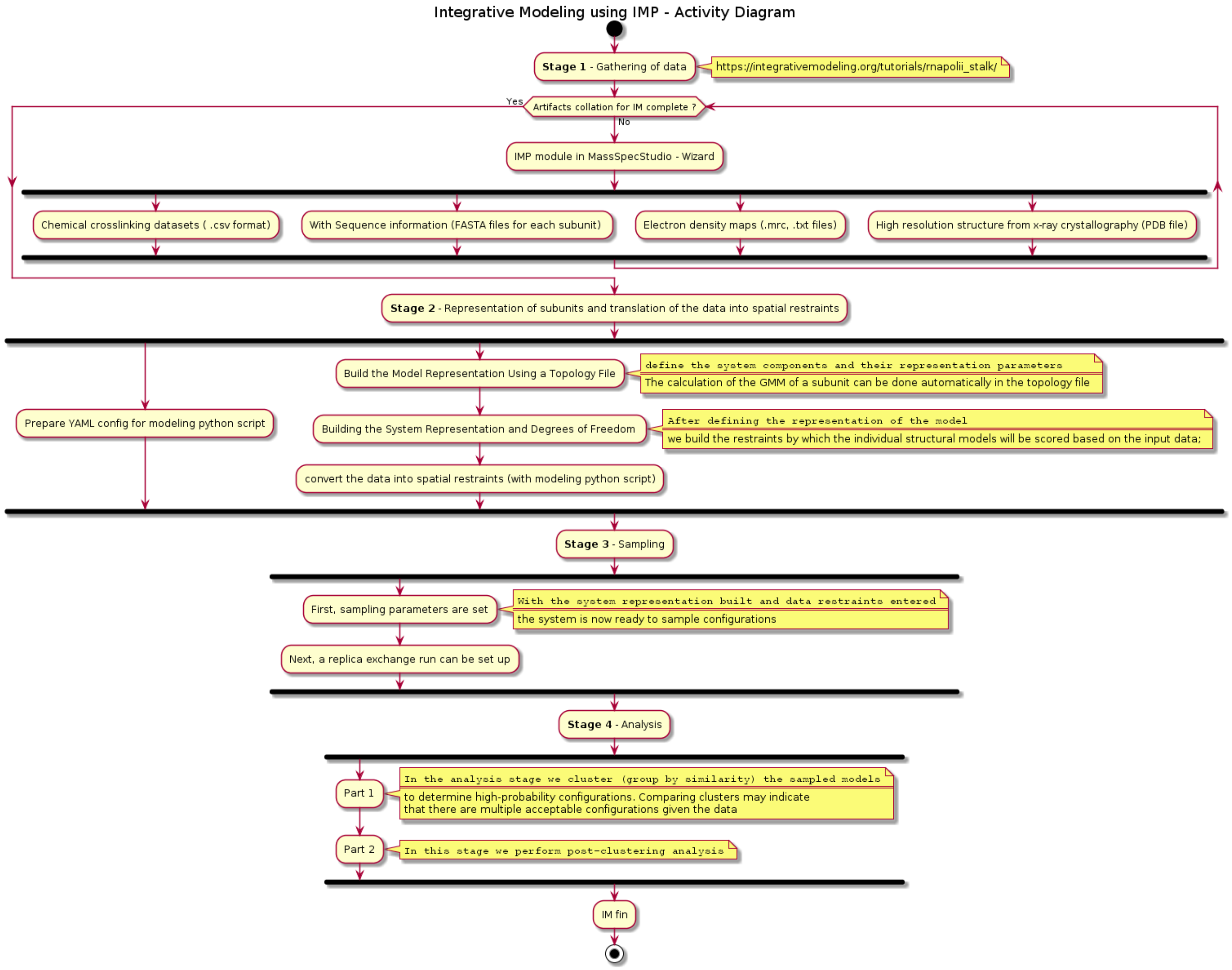

Activity Diagram

- Stage 1 – Gathering data
- Stage 2 – Representation of subunits and translation of the data into spatial restraints
- Stage 3 – Sampling
- Stage 4 - Analysis

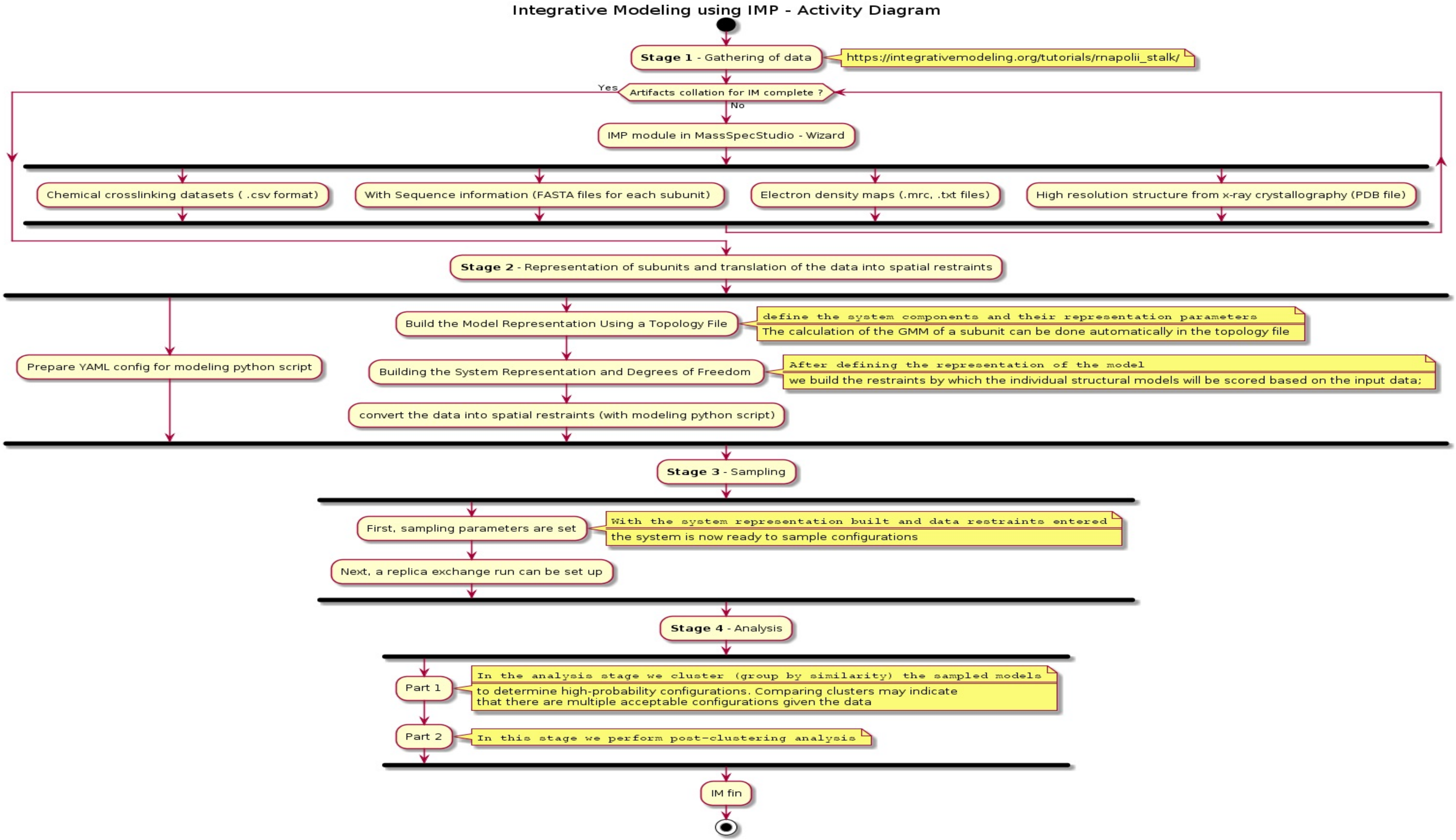
Integrative Modeling using IMP - Activity Diagram
Stage 1 - Gathering of data
https://integrativemodeling.org/tutorials/rnapolii_stalk/
Artifacts collation for IM complete ?
Yes
No
IMP module in MassSpecStudio - Wizard
Chemical crosslinking datasets (.csv format)
With Sequence information (FASTA files for each subunit)
Electron density maps (.mrc, .txt files)
High resolution structure from x-ray crystallography (PDB file)
Stage 2 - Representation of subunits and translation of the data into spatial restraints
Build the Model Representation Using a Topology File
define the system components and their representation parameters
The calculation of the GMM of a subunit can be done automatically in the topology file
Prepare YAML config for modeling python script
Building the System Representation and Degrees of Freedom
After defining the representation of the model
we build the restraints by which the individual structural models will be scored based on the input data;
convert the data into spatial restraints (with modeling python script)
Stage 3 - Sampling
First, sampling parameters are set
With the system representation built and data restraints entered
the system is now ready to sample configurations
Next, a replica exchange run can be set up
Stage 4 - Analysis
Part 1
In the analysis stage we cluster (group by similarity) the sampled models
to determine high-probability configurations. Comparing clusters may indicate
that there are multiple acceptable configurations given the data
Part 2
In this stage we perform post-clustering analysis
IM fin

Overview

- Create a new Integrative modeling project
  - Add Proteins.
  - Add Protein Topology
  - Add Link Data
  - HX-XL Classification
  - Configure IMP
- Amendments
  - Adjustments to existing IMP project.
- Deployment of the Project
  - Perform a modeling run.

Create a new Integrative modeling project

- Familiarize yourself with the IMProv wizard steps.
- The goal here is to produce the Topology and ConfigImp.yaml files.
- Pull together the various raw data files needed for the modeling run.
- Obtain the python driver script that reads the ConfigImp.yaml file
- Understand the folder structure of the export bundle and where the files reside.

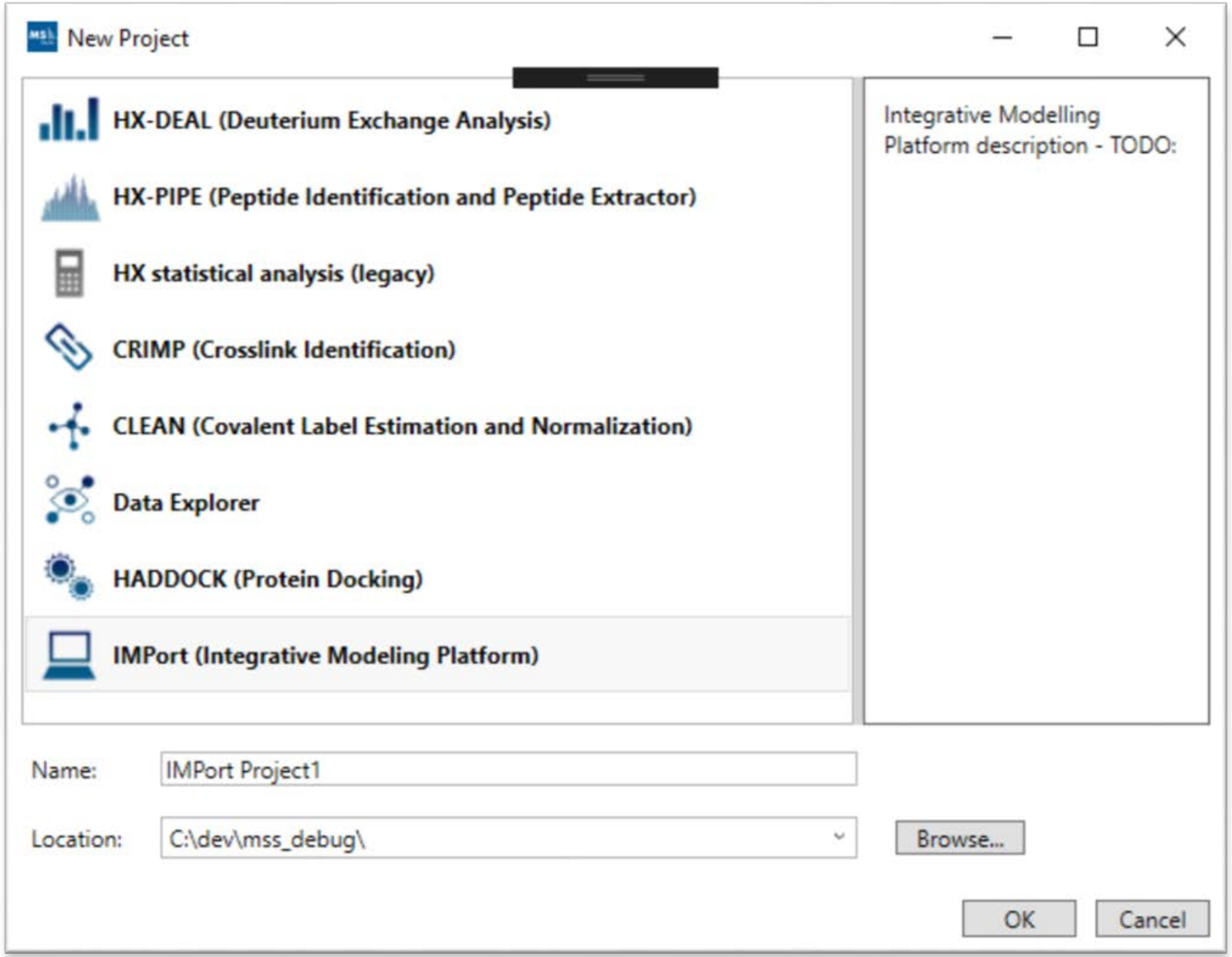

New Project

IMProv (Integrative Modeling Platform)

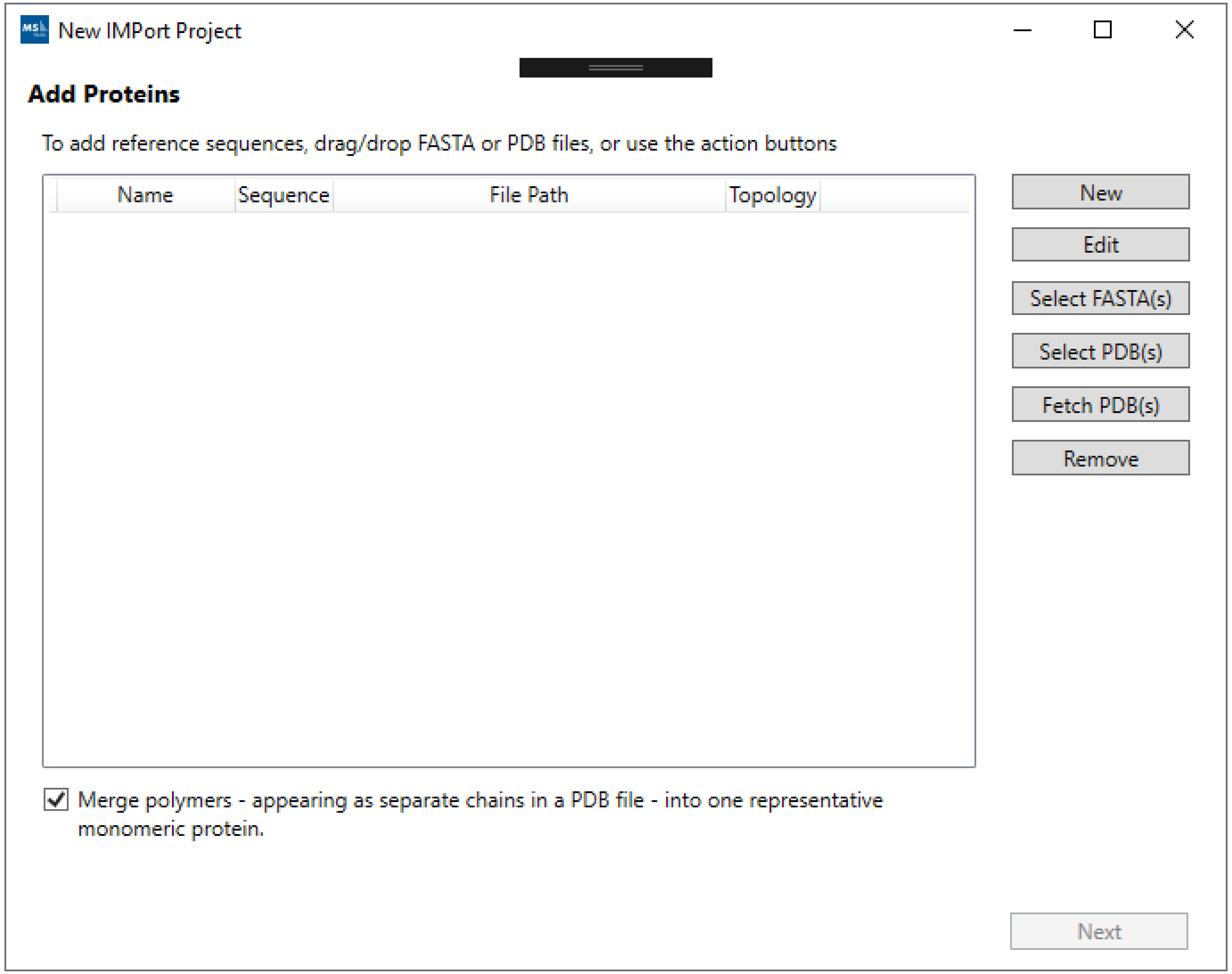

Wizard Steps

- **Add Proteins.**
- Add Protein Topology
- Add Link Data
- HX-XL Classification
- Configure IMP

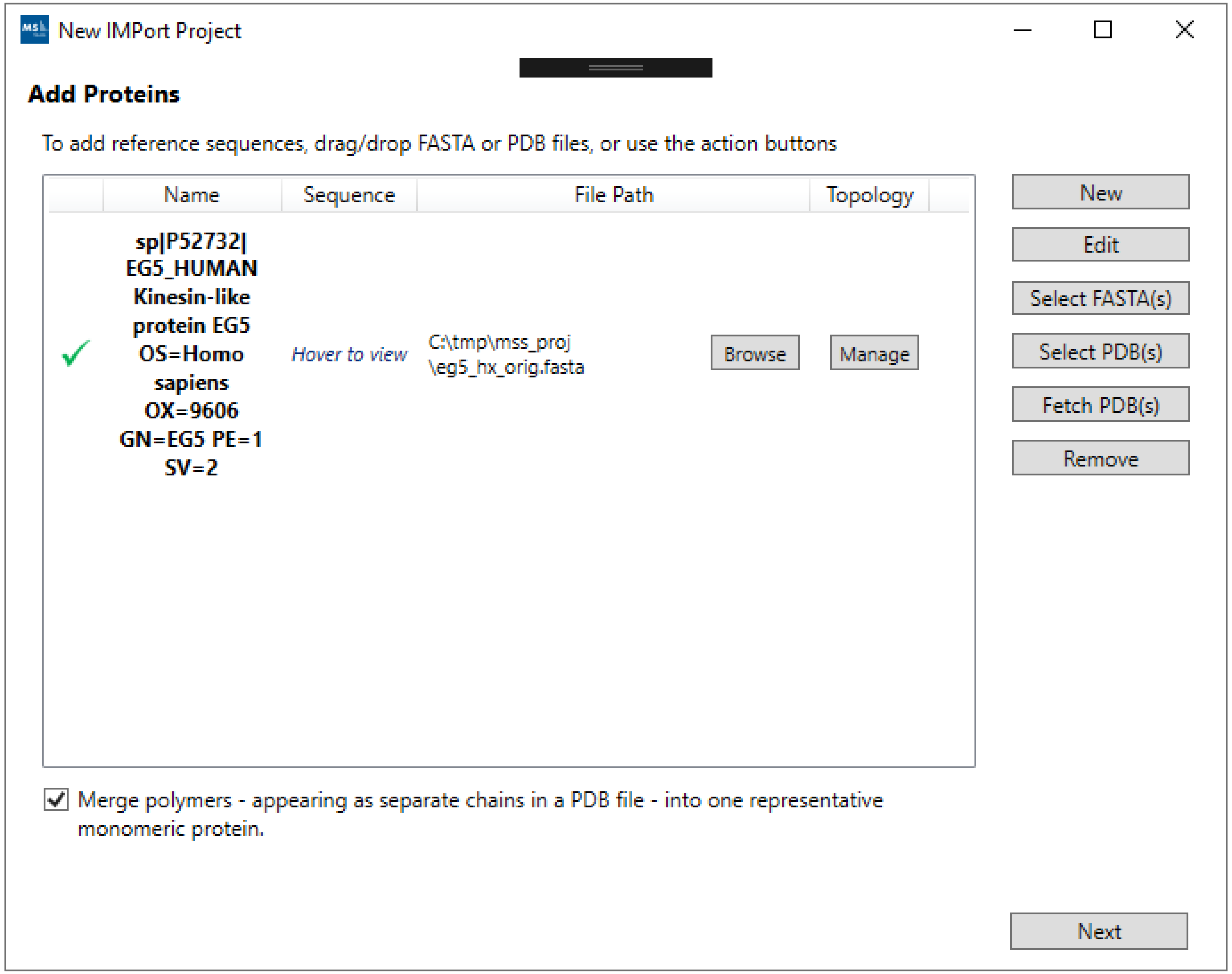

Wizard Steps

- **Add Proteins.**
- Add Protein Topology
- Add Link Data
- HX-XL Classification
- Configure IMP

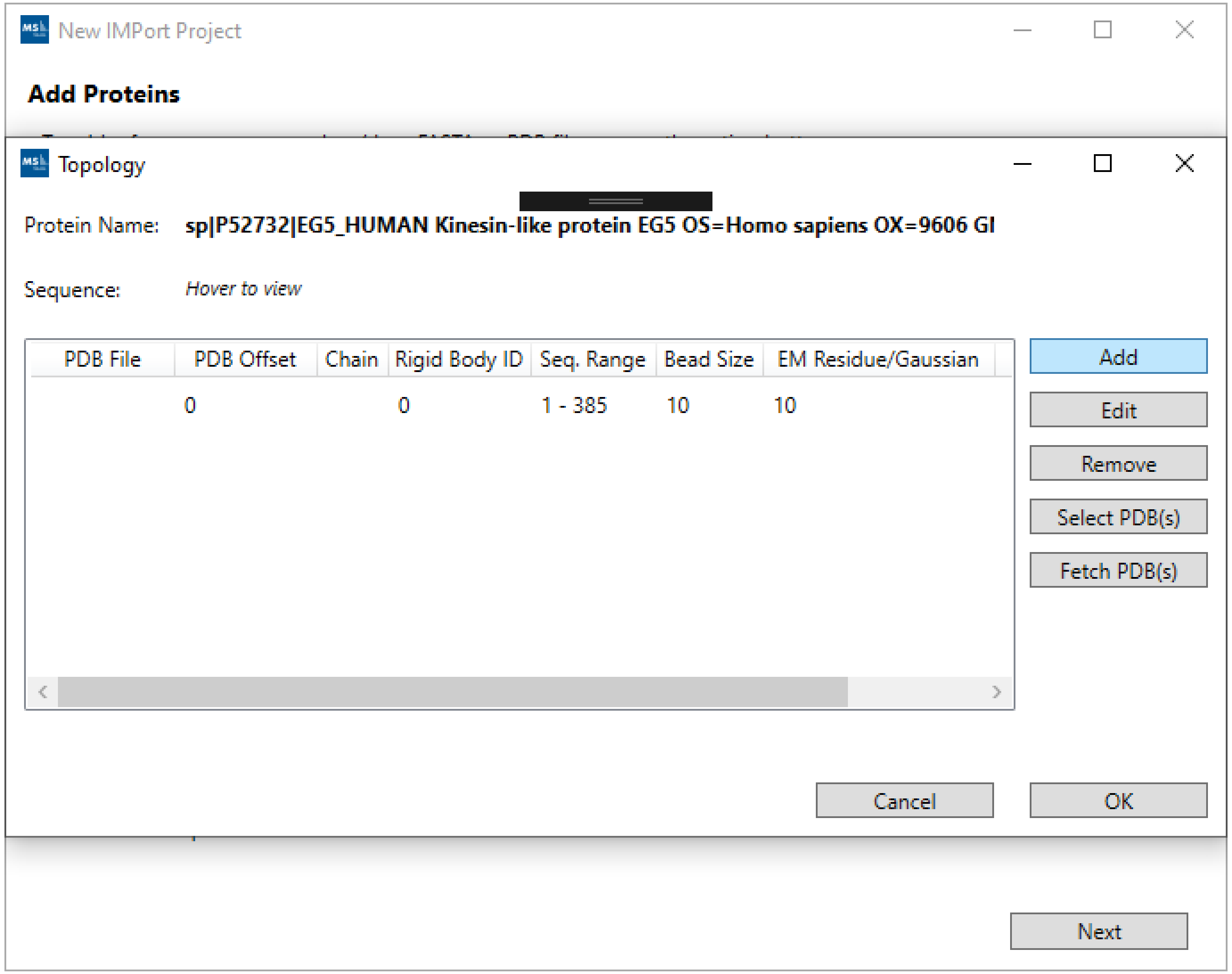

Wizard Steps

- Add Proteins.
- **Add Protein Topology**
- Add Link Data
- HX-XL Classification
- Configure IMP

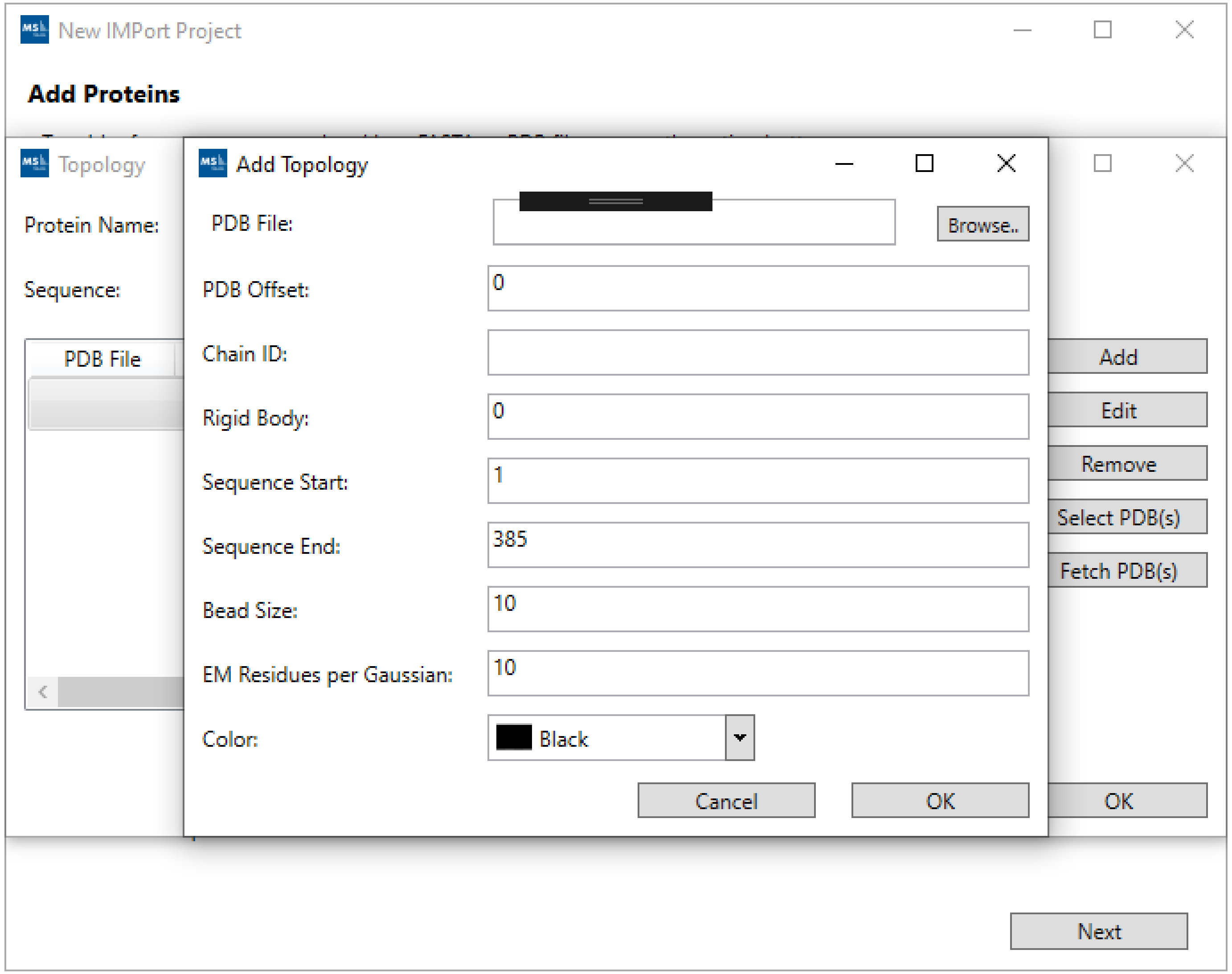

Wizard Steps

- Add Proteins.
- Add Protein Topology
- Add Link Data
- HX-XL Classification
- Configure IMP

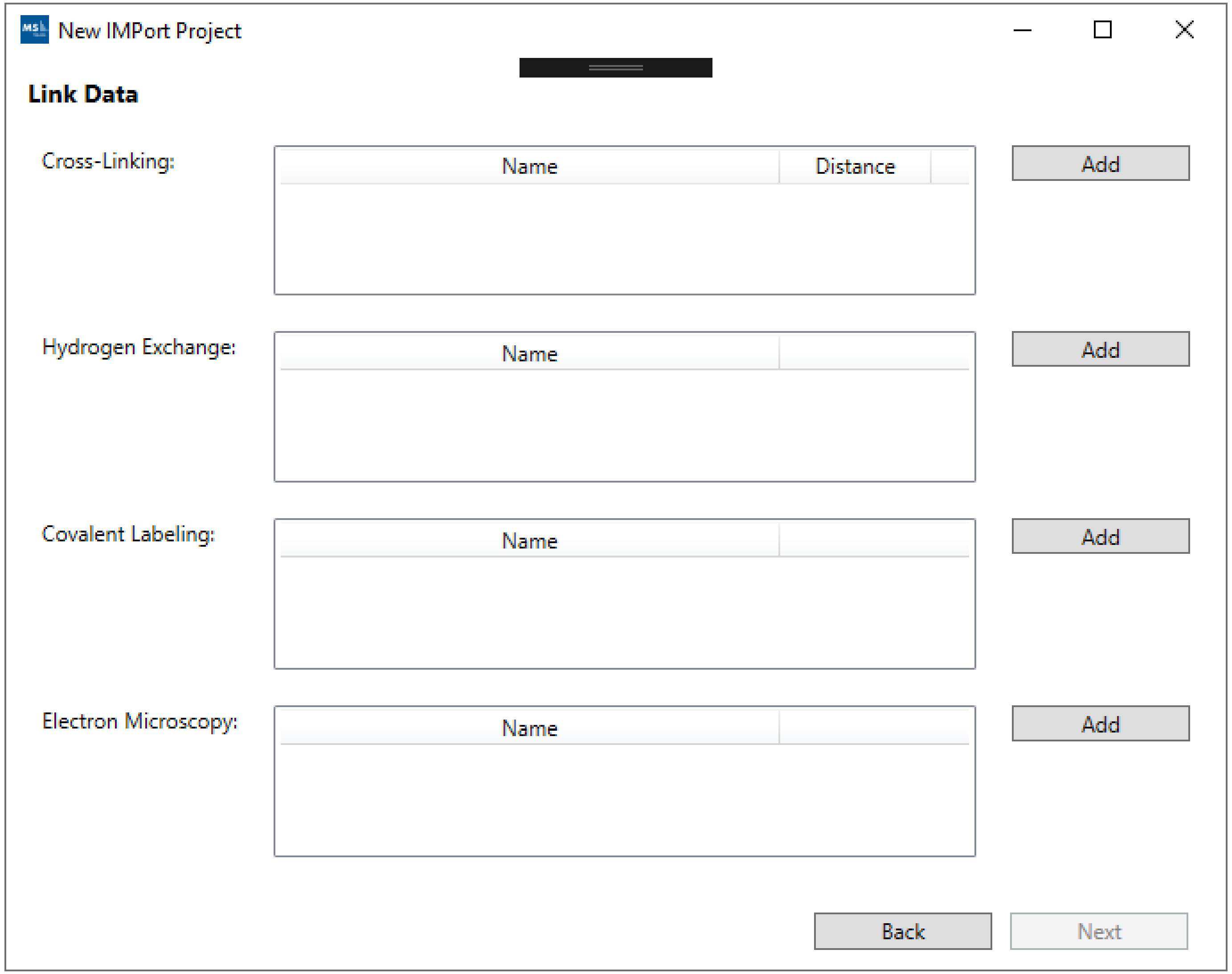

Wizard Steps

- Add Proteins.
- Add Protein Topology
- **Add Link Data**
- HX-XL Classification
- Configure IMP

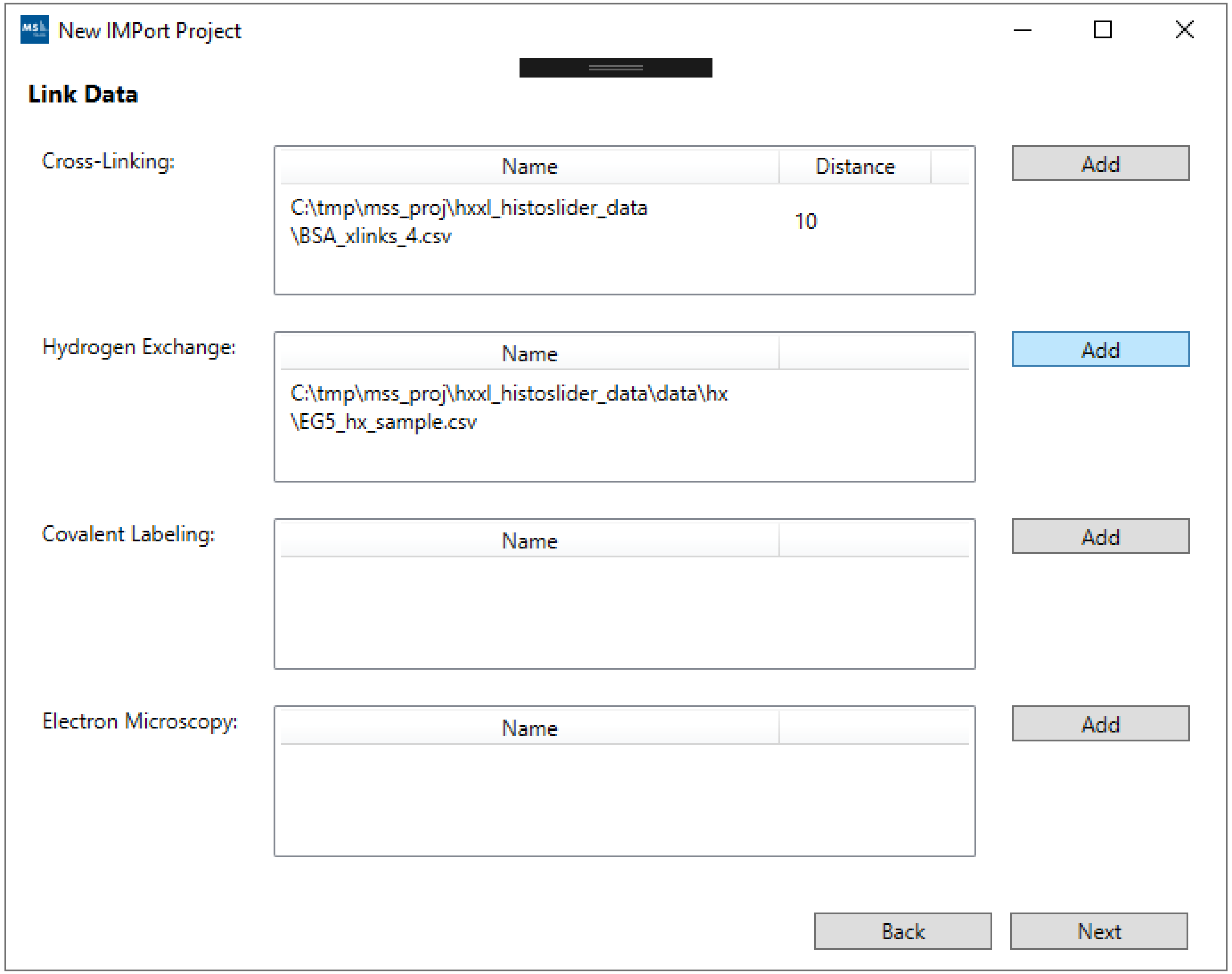

Wizard Steps

- Add Proteins.
- Add Protein Topology
- **Add Link Data**
- HX-XL Classification
- Configure IMP

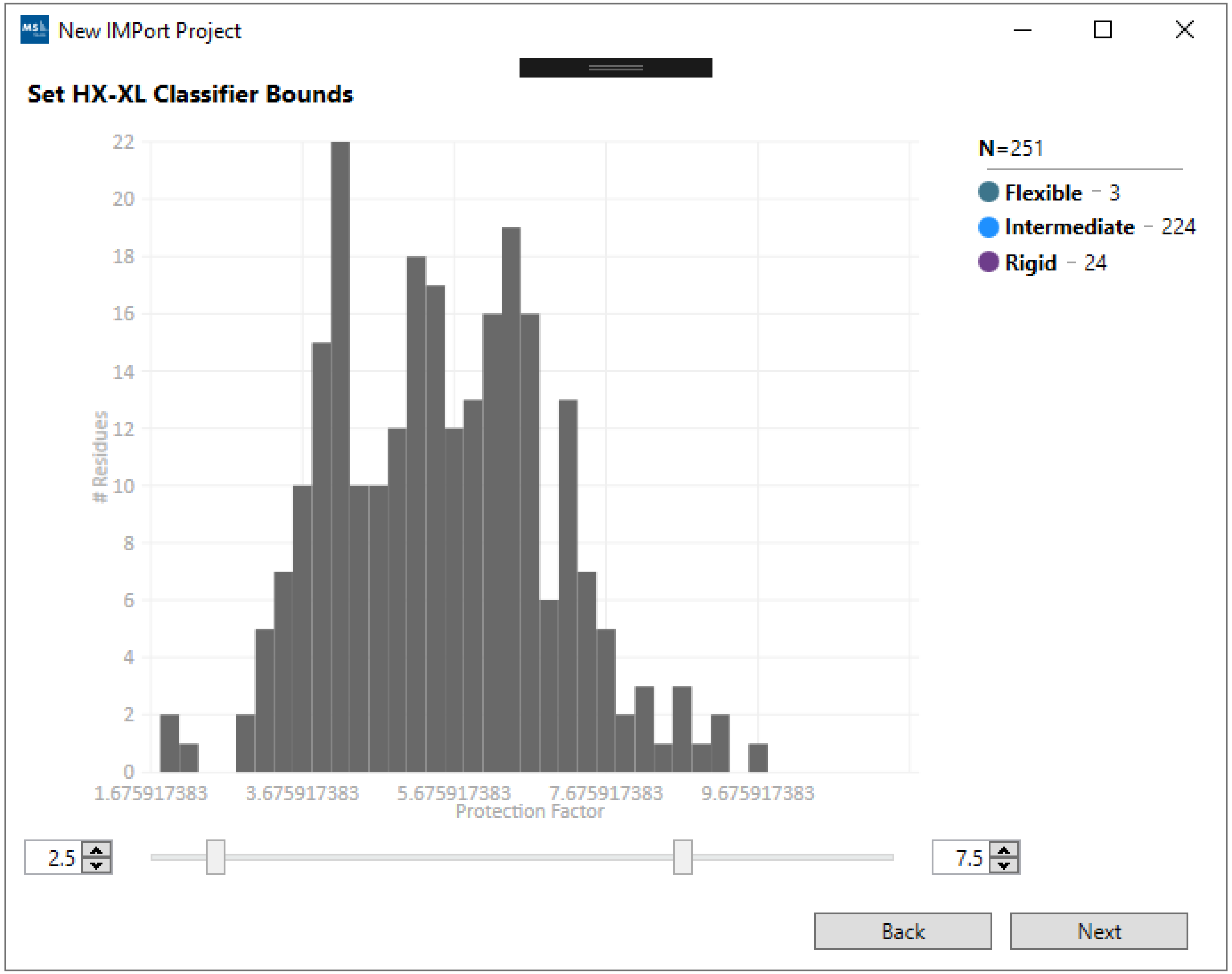

Wizard Steps

- Add Proteins.
- Add Protein Topology
- Add Link Data
- **HX-XL Classification**
- Configure IMP

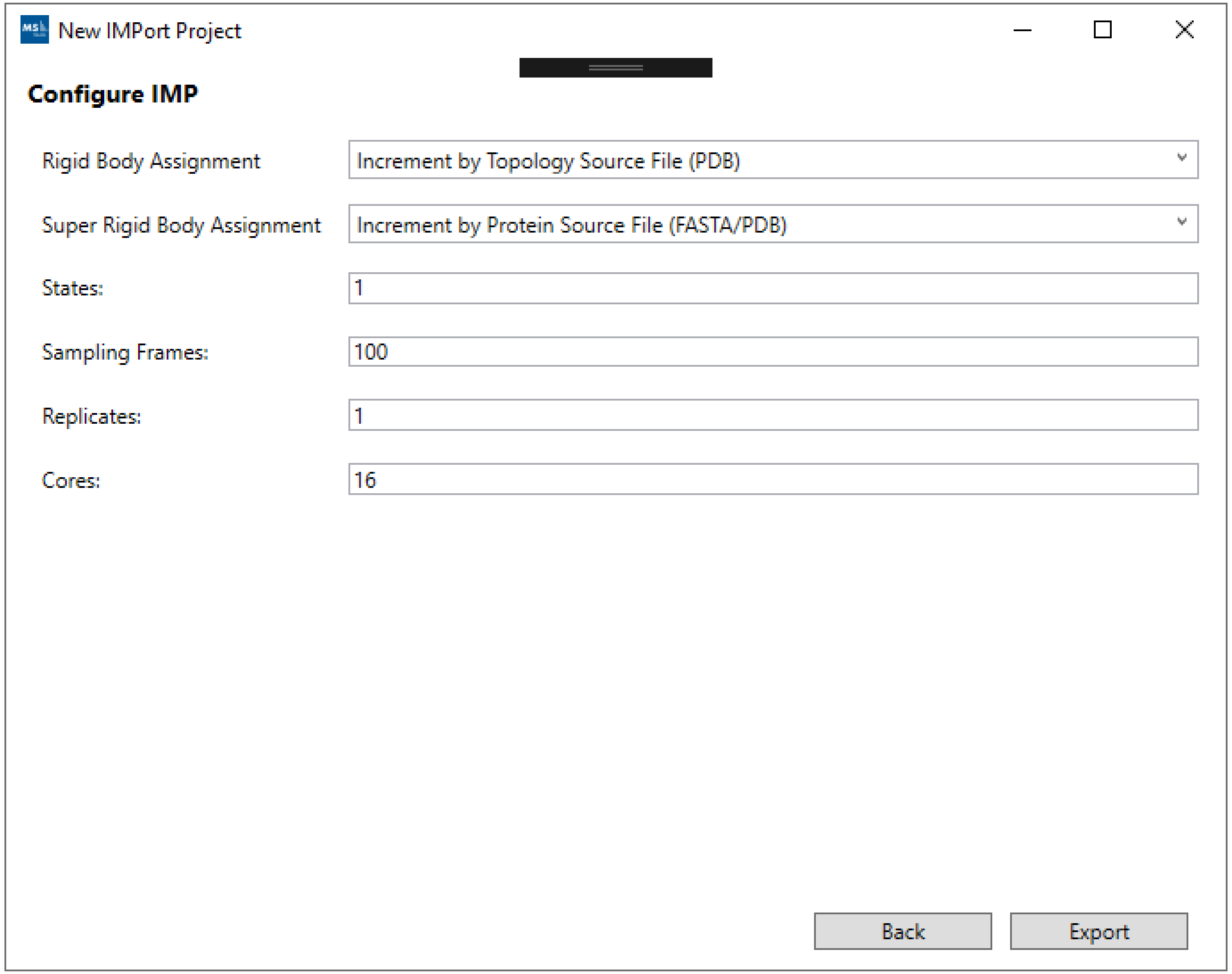

Wizard Steps

- Add Proteins.
- Add Protein Topology
- Add Link Data
- HX-XL Classification
- **Configure IMP**

IMProv Data File and Script Bundle - Export

- Generate the IMProv Export Bundle.
- Navigate the folders.
- Review the Topology and ConfigImp.yaml files.

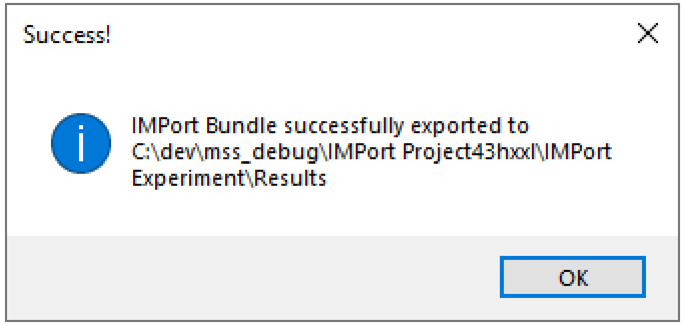

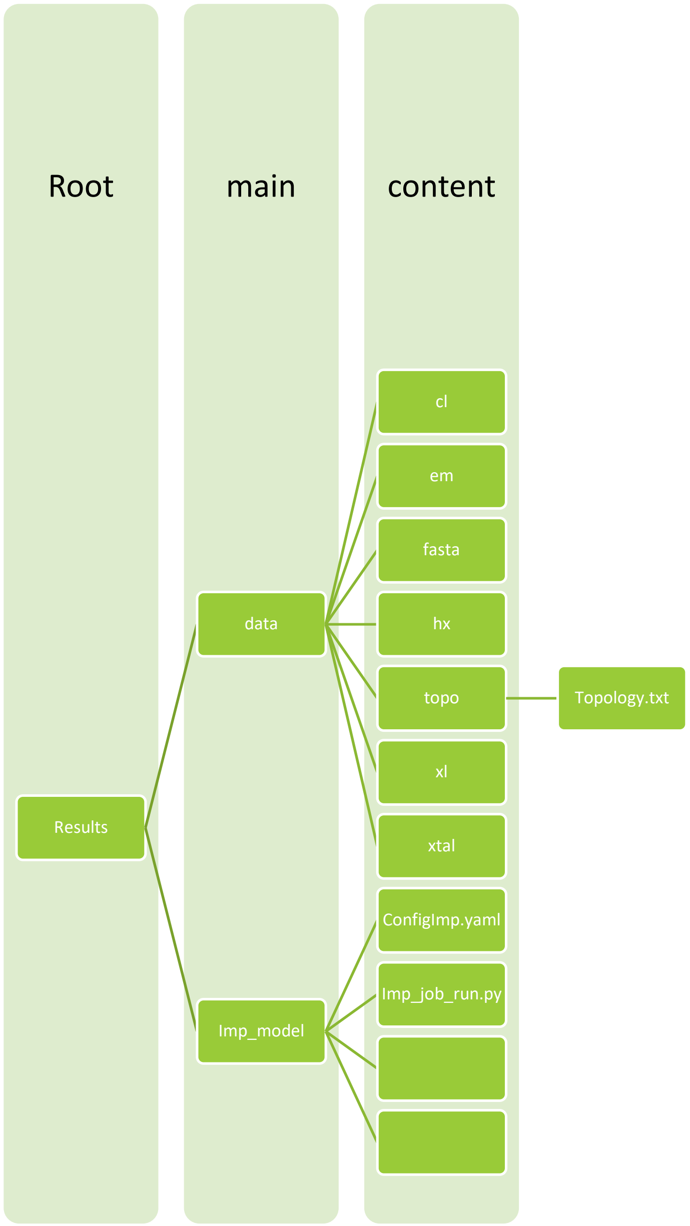

Folder Hierarchy

data

- cl => crosslink
- em => electron density maps
- fasta => protein amino acid sequence
- hx => hydrogen exchange
- topo => topology file
- xl => cross linking
- xtal => xray-crystallography

imp_model

config and driver scripts

Completion of IMProv wizard steps

- Review output generated.
- Prepare output for deployment.

Amendments

- Modifying the Topology and ConfigImp.yaml is an iterative process.
- Re-open an existing IMProv project and make the edits you require.
- In the final step you can perform the export again to capture the updates.

IMProv export bundle

- Review Topology.txt and ConfigImp.yaml
- Review python driver scripts

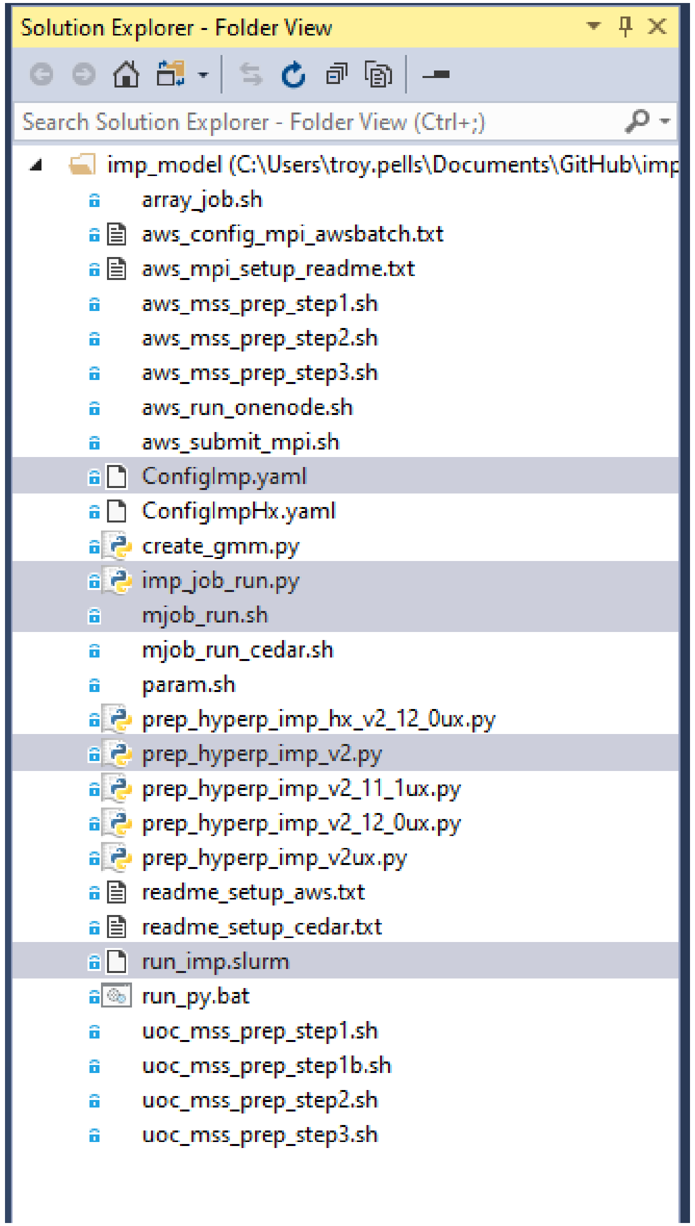

ConfigImp.yaml and driver scripts

- Sample PRC2 project contains the ConfigImp.yaml and driver scripts
- We also find setup scripts for Cedar and AWS. These are discussed later, when we cover the deployment steps.

ConfigImp.yaml

ConfigImp.yaml


```yaml
title: PRC2 with HX and EM
date: 2019-07-10T12:10:31.1913934-06:00
cores: 2
replicates: 1
states: 1
sampling_frame: 20
output_dir: ./imp_model
data_directory: ../
topology_file: Topology.txt
target_gmm_file: gmm_file_ouput.txt
crosslinkdb:
- refid: PRC2_DSS_unprotected.csv
- refid: PRC2_DSS_standard.csv
- refid: PRC2_DSS_protected.csv
- refid: PRC2_BS3_standard.csv
- refid: PRC2_BS3_unprotected.csv
- refid: PRC2_BS3_protected.csv
xl_groupA:
- refid: PRC2_DSS_unprotected.csv
  length: 21.0
  slope: 0.01
  resolution: 1.0
  label: PRC2_DSS_unprotected
  weight: 1.0
  crosslink_distance: 20.0
- refid: PRC2_DSS_standard.csv
```

```yaml
xl_dbA:
- refid: PRC2_DSS_unprotected.csv
  set_protein1_key: Protein 1
  set_protein2_key: Protein 2
  set_site_pairs_key: Selected Sites
  set_unique_id_key: Peptide ID
- refid: PRC2_DSS_standard.csv
  set_protein1_key: Protein 1
  set_protein2_key: Protein 2
  set_site_pairs_key: Selected Sites
  set_unique_id_key: Peptide ID
- refid: PRC2_DSS_protected.csv
  set_protein1_key: Protein 1
  set_protein2_key: Protein 2
  set_site_pairs_key: Selected Sites
  set_unique_id_key: Peptide ID
- refid: PRC2_BS3_standard.csv
  set_protein1_key: Protein 1
  set_protein2_key: Protein 2
  set_site_pairs_key: Selected Sites
  set_unique_id_key: Peptide ID
- refid: PRC2_BS3_unprotected.csv
  set_protein1_key: Protein 1
  set_protein2_key: Protein 2
  set_site_pairs_key: Selected Sites
  set_unique_id_key: Peptide ID
- refid: PRC2_BS3_protected.csv
  set_protein1_key: Protein 1
  set_protein2_key: Protein 2
  set_site_pairs_key: Selected Sites
  set_unique_id_key: Peptide ID
degree_of_freedom:
  max_rb_trans: 4.0
  max_rb_rot: 0.3
  max_bead_trans: 4.0
  max_srb_trans: 4.0
  max_srb_rot: 0.3
```

imp_job_run.py and **mjob_run.sh** scripts

Topology.txt | run_imp.slurm | prep_hyperp_imp_v2.py | mjob_run.sh | imp_job_run.py | ConfigImp.yaml

```python
#!/usr/bin/python3

import os, subprocess, platform, argparse

# default paths to anaconda and data roots
ANACONDA_DIR = os.path.join("C:\\", "Apps", "Anaconda3") if platform.system() == "Windows" else os.path.join("~", "anaconda3")
DEFAULT_COUNT, DEFAULT_NAME, DEFAULT_CONFIG = 1, "DemoImpModel", "ConfigImp.yaml"

# parse args for path replacements & args for the job start command execution
parser = argparse.ArgumentParser()
parser.add_argument("--anaconda_dir", type=str, help="path to the root directory of your Anaconda installation")
parser.add_argument("--count", type=int, help="count variable for IMP")
parser.add_argument("--name", type=str, help="Name of job")
parser.add_argument("--config", type=str, help="config file name (within imp_model directory)")
parser.add_argument("--output_file", type=str, help="file to redirect imp script execution into, rather than stdout")
args = parser.parse_args()

if args.anaconda_dir is not None:
    ANACONDA_DIR = args.anaconda_dir

outfile = args.output_file if args.output_file is not None else "prep_hyperp_imp_v2_trace.txt"
with open(outfile, 'w') as f:
    subprocess.run(
        "{0} prep_hyperp_imp_v2.py --count={1} --name={2} --config={3}".format(
            os.path.join(ANACONDA_DIR, "python.exe"),
            args.count  if args.count  is not None else DEFAULT_COUNT,
            args.name   if args.name   is not None else DEFAULT_NAME,
            args.config if args.config is not None else DEFAULT_CONFIG,
        ),
        stdout=f,
        stderr=(subprocess.STDOUT)
    )
```

Topology.txt | run_imp.slurm | prep_hyperp_imp_v2.py | mjob_run.sh | imp_job_run.py | ConfigImp.yaml

```bash
# !/bin/bash -x

# usage: mjob_run.sh 6
# first param is the replicate number
num_repl=$1
repl_name=imp_model_$1
echo "replicates number:"$repl_name
#set defaults for cores and replicates
export cores="16"
#cd ../
#cp -R imp_model imp_model_2
#cp -R imp_model imp_model_3
#sed -e 's/:[^:\/\/]/="/g;s/$/"/g;s/ *=/=/g' ConfigImp.yaml > param.sh
cat ConfigImp.yaml | grep -e cores | sed -e 's/:[^:\/\/]/="/g;s/$/"/g;s/ *=/=/g' | sed -e 's/ //g' | sed -e 's/^/export /g' > para
chmod 755 param.sh
#source the env vars from param.sh as these are exported
. param.sh
echo "cores:"$cores
# construct slurm file
cat << EOF > param_prep.slurm
#!/bin/bash
# example slurm job script setup to run on for example UoC ARC
#SBATCH --job-name=SLURM_imp
#SBATCH --nodes=1
#SBATCH --ntasks-per-node=$cores
#SBATCH --cpus-per-task=1
#SBATCH --mem=32G
#SBATCH --time=00:30:00
#SBATCH --partition=cpu2019




# mpiexec python prep_hyperp_imp_v2ux.py --count=1 --name=DemoImpModel --config=ConfigImp.yaml
srun python prep_hyperp_imp_v2ux.py --count=1 --name=DemoImpModel --config=ConfigImp.yaml

echo "Job Finished"
EOF
# nix line ending and not wnd
sed -e "s/\r//" param_prep.slurm > run_imp.slurm
#clean up intermediate files
rm -rf param_prep.slurm
rm -rf param.sh
# from imp_model folder we clone the driver scripts
cd ../
cp -R imp_model $repl_name
cd $repl_name
#sbatch run_imp.slurm
#squeue -u john.smith
```

prep_hyperp_imp_v2.py script content

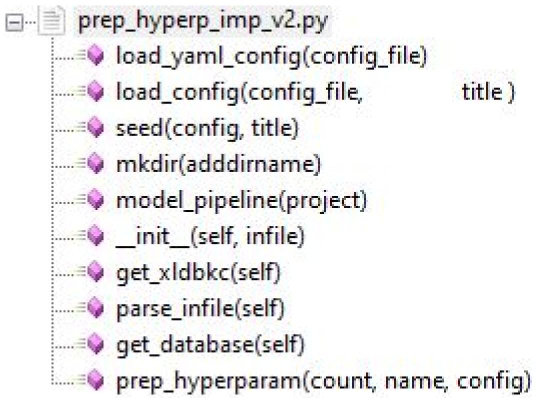

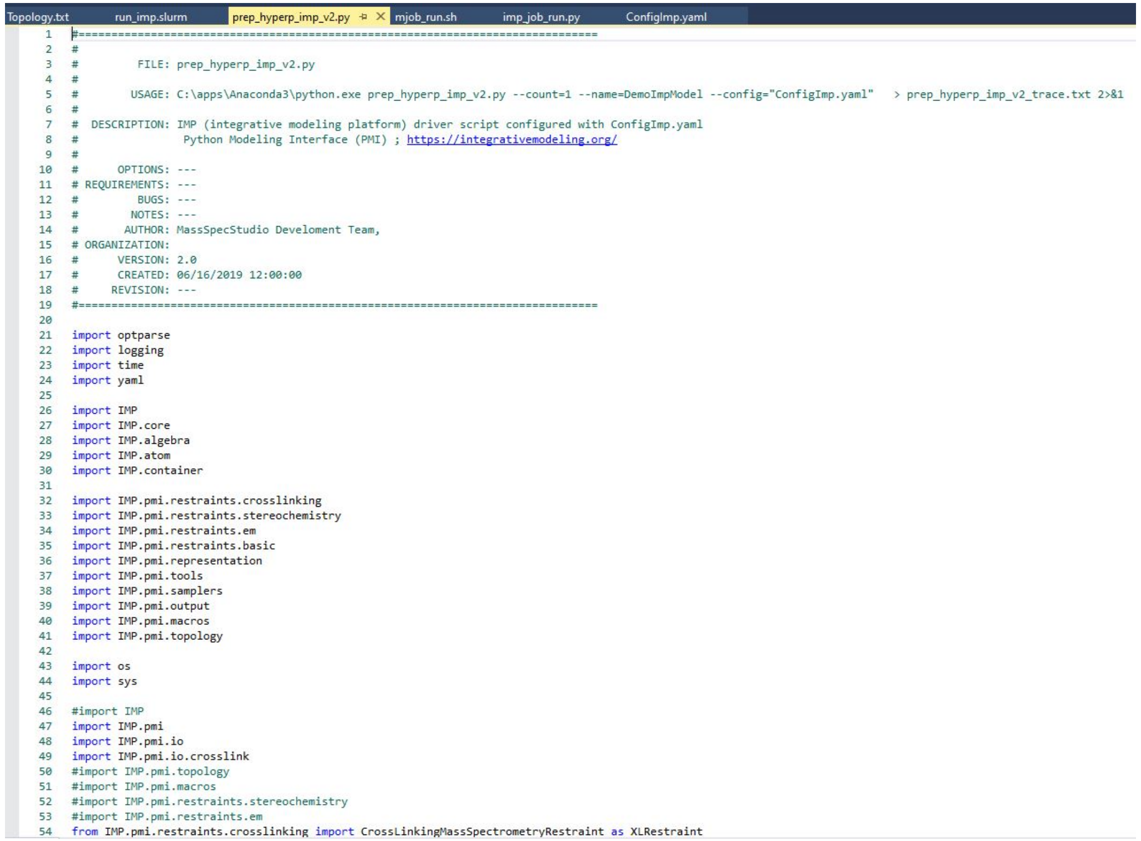

Topology.txt | run_imp.slurm | prep_hyperp_imp_v2.py | mjob_run.sh | imp_job_run.py | ConfigImp.yaml

```
#==============================================================================
#
#         FILE: prep_hyperp_imp_v2.py
#
#        USAGE: C:\apps\Anaconda3\python.exe prep_hyperp_imp_v2.py --count=1 --name=DemoImpModel --config="ConfigImp.yaml"   > prep_hyperp_imp_v2_trace.txt 2>&1
#
#  DESCRIPTION: IMP (integrative modeling platform) driver script configured with ConfigImp.yaml
#                Python Modeling Interface (PMI) ; https://integrativemodeling.org/
#
#      OPTIONS: ---
# REQUIREMENTS: ---
#         BUGS: ---
#        NOTES: ---
#       AUTHOR: MassSpecStudio Develoment Team,
# ORGANIZATION:
#      VERSION: 2.0
#      CREATED: 06/16/2019 12:00:00
#     REVISION: ---
#==============================================================================

import optparse
import logging
import time
import yaml

import IMP
import IMP.core
import IMP.algebra
import IMP.atom
import IMP.container

import IMP.pmi.restraints.crosslinking
import IMP.pmi.restraints.stereochemistry
import IMP.pmi.restraints.em
import IMP.pmi.restraints.basic
import IMP.pmi.representation
import IMP.pmi.tools
import IMP.pmi.samplers
import IMP.pmi.output
import IMP.pmi.macros
import IMP.pmi.topology

import os
import sys

#import IMP
import IMP.pmi
import IMP.pmi.io
import IMP.pmi.io.crosslink
#import IMP.pmi.topology
#import IMP.pmi.macros
#import IMP.pmi.restraints.stereochemistry
#import IMP.pmi.restraints.em
from IMP.pmi.restraints.crosslinking import CrossLinkingMassSpectrometryRestraint as XLRestraint
```

load_config

- prep_hyperp_imp_v2.py
  - load_yaml_config(config_file)
  - load_config(config_file, title)
  - seed(config, title)
  - mkdir(adddirname)
  - model_pipeline(project)
  - __init__(self, infile)
  - get_xldbkc(self)
  - parse_infile(self)
  - get_database(self)
  - prep_hyperparam(count, name, config)

```python
def load_config(config_file,
                title ):
    """
    Parses a project.yaml file and uses the contents to
    set the current execution context.

    Optionally injects additional values
    https://martin-thoma.com/configuration-files-in-python/

    https://github.com/beetbox/confuse
    https://hackersandslackers.com/simplify-your-python-projects-configuration/

    """

    logging.info('config filename %s!' % config_file)
    #obj = yaml.safe_load(config_file)
    cfg = load_yaml_config(config_file)
    for section in cfg:
        logging.info(section + ' %s!' % cfg[section])

        if section == "xl_groupA":
            #
            for i in cfg[section]:
                logging.info(i)
                for k, v in i.items():
                    logging.info(k +': %s!' % v)

        if section == "xl_dbA":
            #
            for i in cfg[section]:
                logging.info(i)
                for k, v in i.items():
                    logging.info(k +': %s!' % v)

        if section == "crosslinkdb":
            #
            for i in cfg[section]:
                logging.info(i)
                for k, v in i.items():
                    logging.info(k +': %s!' % v)


        if section == "degree_of_freedom":
            #logging.info( subxl_group + ' %s!' % cfg[section])
            for i in cfg[section]:
                logging.info(i +': %s!' % cfg[section][i])

    #print(cfg['topology_file'])
    #print(cfg['title'])
    logging.info('given topology_file %s!' % cfg['topology_file'])
    logging.info('given title %s!' % cfg['title'])



    # perform pipeline setup
    model_pipeline(cfg)



def seed(config, title):
```

Monte-Carlo Sampling

- prep_hyperp_imp_v2.py
  - load_yaml_config(config_file)
  - load_config(config_file, title)
  - seed(config, title)
  - mkdir(adddirname)
  - model_pipeline(project)
  - __init__(self, infile)
  - get_xldbkc(self)
  - parse_infile(self)
  - get_database(self)
  - prep_hyperparam(count, name, config)

```
#--------------------------
# Monte-Carlo Sampling
#--------------------------

#--------------------------
# Set MC Sampling Parameters
#--------------------------
#num_frames = 20000
num_frames = 50
#if '--test' in sys.argv: num_frames=100
num_mc_steps = 10

logging.info('set states %s!' % project["states"])
logging.info('set sampling_frame %s!' % project["sampling_frame"])
logging.info('set num_frames %s!' % num_frames)

logging.info('set output_dir %s!' % project["output_dir"])
logging.info('set num_mc_steps %s!' % num_mc_steps)



#TODO: add config setup for these fixed values
logging.info('set monte_carlo_temperature=1.0')
logging.info('set simulated_annealing=True')
logging.info('set simulated_annealing_minimum_temperature=1.0')
logging.info('set simulated_annealing_maximum_temperature=2.5')
logging.info('set simulated_annealing_minimum_temperature_nframes=200')
logging.info('set simulated_annealing_maximum_temperature_nframes=20')
logging.info('set replica_exchange_minimum_temperature=1.0')
logging.info('set replica_exchange_maximum_temperature=2.5')
logging.info('set number_of_best_scoring_models=0')
logging.info('set monte_carlo_steps %s!' % num_mc_steps)
logging.info('set number_of_frames %s!' % num_frames)
logging.info('set global_output_directory %s!' % project["output_dir"])



# https://integrativemodeling.org/2.10.1/doc/ref/classIMP_1_1pmi_1_1macros_1_1ReplicaExchange0.html#a239c4009cc04c70236730479f9f79744
# This object defines all components to be sampled as well as the sampling protocol
mc1=IMP.pmi.macros.ReplicaExchange0(mdl,
                                    root_hier=root_hier,
                                    monte_carlo_sample_objects=dof.get_movers(),
                                    output_objects=outputobjects,
                                    crosslink_restraints=xl_rests,    # allows XLs to be drawn in the RMF files
                                    monte_carlo_temperature=1.0,
                                    simulated_annealing=True,
                                    simulated_annealing_minimum_temperature=1.0,
                                    simulated_annealing_maximum_temperature=2.5,
                                    simulated_annealing_minimum_temperature_nframes=200,
                                    simulated_annealing_maximum_temperature_nframes=20,
                                    replica_exchange_minimum_temperature=1.0,
                                    replica_exchange_maximum_temperature=2.5,
                                    number_of_best_scoring_models=0,
                                    monte_carlo_steps=num_mc_steps, #keep at 10
                                    number_of_frames=num_frames,
                                    global_output_directory=project["output_dir"],
                                    test_mode=False)

# start sampling
```

ReplicaExchange

- prep_hyperp_imp_v2.py
  - load_yaml_config(config_file)
  - load_config(config_file, title)
  - seed(config, title)
  - mkdir(adddirname)
  - model_pipeline(project)
  - __init__(self, infile)
  - get_xldbkc(self)
  - parse_infile(self)
  - get_database(self)
  - prep_hyperparam(count, name, config)

```python
#TODO: add config setup for these fixed values
logging.info('set monte_carlo_temperature=1.0')
logging.info('set simulated_annealing=True')
logging.info('set simulated_annealing_minimum_temperature=1.0')
logging.info('set simulated_annealing_maximum_temperature=2.5')
logging.info('set simulated_annealing_minimum_temperature_nframes=200')
logging.info('set simulated_annealing_maximum_temperature_nframes=20')
logging.info('set replica_exchange_minimum_temperature=1.0')
logging.info('set replica_exchange_maximum_temperature=2.5')
logging.info('set number_of_best_scoring_models=0')
logging.info('set monte_carlo_steps %s!' % num_mc_steps)
logging.info('set number_of_frames %s!' % num_frames)
logging.info('set global_output_directory %s!' % project["output_dir"])



# https://integrativemodeling.org/2.10.1/doc/ref/classIMP_1_1pmi_1_1macros_1_1ReplicaExchange0.html#a239c4009cc04c70236730479f9f79744
# This object defines all components to be sampled as well as the sampling protocol
mc1=IMP.pmi.macros.ReplicaExchange0(mdl,
                                    root_hier=root_hier,
                                    monte_carlo_sample_objects=dof.get_movers(),
                                    output_objects=outputobjects,
                                    crosslink_restraints=xl_rests,    # allows XLs to be drawn in the RMF files
                                    monte_carlo_temperature=1.0,
                                    simulated_annealing=True,
                                    simulated_annealing_minimum_temperature=1.0,
                                    simulated_annealing_maximum_temperature=2.5,
                                    simulated_annealing_minimum_temperature_nframes=200,
                                    simulated_annealing_maximum_temperature_nframes=20,
                                    replica_exchange_minimum_temperature=1.0,
                                    replica_exchange_maximum_temperature=2.5,
                                    number_of_best_scoring_models=0,
                                    monte_carlo_steps=num_mc_steps, #keep at 10
                                    number_of_frames=num_frames,
                                    global_output_directory=project["output_dir"],
                                    test_mode=False)

# start sampling
mc1.execute_macro()

#logging.info("GEMT", gemt.evaluate());
#logging.info("XL1", xl1.evaluate(), xl2.evaluate());
for i in range(len(xlList) ):
    logging.info(xlList[i].evaluate())
logging.info("EV", ev.evaluate());
logging.info("CR", cr.evaluate());
```

topology.txt data dictionary

A	B	C
**empty_field**		
**molecule_name**	EZH2	*EED*
**color**	black	*black*
**fasta_fn**	prc2_pentamer.fasta	*prc2_pentamer.fas*
**fasta_id**	EZH2	*EED*
**pdb_fn**	5hyn_ABC.pdb	*5hyn_ABC.pdb*
**chain**	A	*B*
**residue_range**	1,END	*1,END*
**pdb_offset**	0	*2*
**bead_size**	10	*10*
**em_residues**	10	*10*
**rigid_body**	1	*1*
**super_rigid_body**	1	*1*
**chain_of_super_rigid_bodies**		
**flags**		

A	B	C	D	E	F	G	H	I	J	K	L	M	N	O
empty_field	molecule_name	color	fasta_fn	fasta_id	pdb_fn	chain	residue_range	pdb_offset	bead_size	em_residues	rigid_body	super_rigid_body	chain_of_super_rigid_bodies	flags
	EZH2	black	prc2_pentamer.fasta	EZH2	5hyn_ABC.pdb	A	1,END	0	10	10	1	1		
	EED	black	prc2_pentamer.fasta	EED	5hyn_ABC.pdb	B	1,END	2	10	10	1	1		
	SUZ12	black	prc2_pentamer.fasta	SUZ12	RBBP4-threaded2yb8_SUZ12-fragment.pdb	B	1,560	0	10	10	2	1		
	SUZ12	black	prc2_pentamer.fasta	SUZ12	5hyn_ABC.pdb	C	561,END	1	10	10	1	1		
	RBBP4	black	prc2_pentamer.fasta	RBBP4	RBBP4-threaded2yb8_SUZ12-fragment.pdb	A	1,END	0	10	10	2	1		
	AEBP2	black	prc2_pentamer.fasta	AEBP2	AEBP2_HMM_A_69-79.pdb	A	1,79	0	10	10	3	2		
	AEBP2	black	prc2_pentamer.fasta	AEBP2	AEBP2_HMM_A_107-117.pdb	A	80,117	0	10	10	4	2		
	AEBP2	black	prc2_pentamer.fasta	AEBP2	AEBP2_HMM_A_136-146.pdb	A	118,146	0	10	10	5	2		
	AEBP2	black	prc2_pentamer.fasta	AEBP2	AEBP2_HMM_A_166-175.pdb	A	147,175	0	10	10	6	2		
	AEBP2	black	prc2_pentamer.fasta	AEBP2	AEBP2_HMM_A_229-243.pdb	A	176,END	0	10	10	7	2		

data folder content

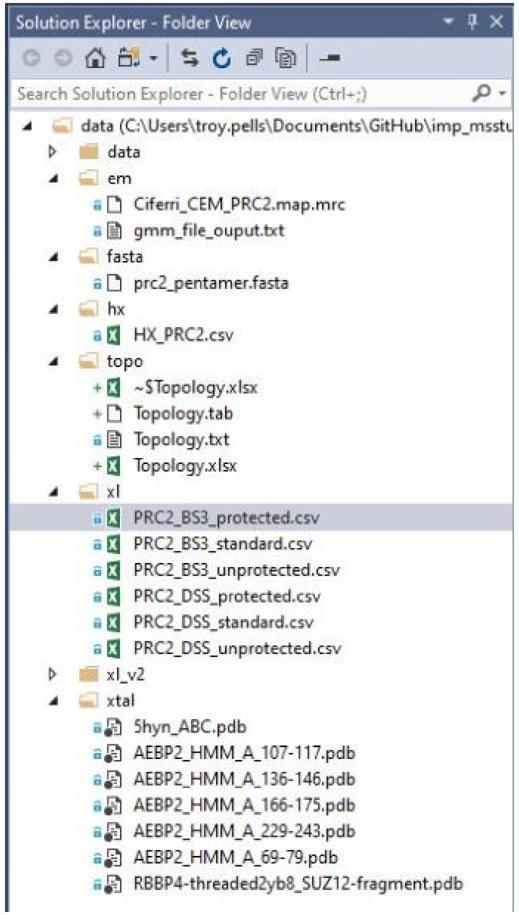

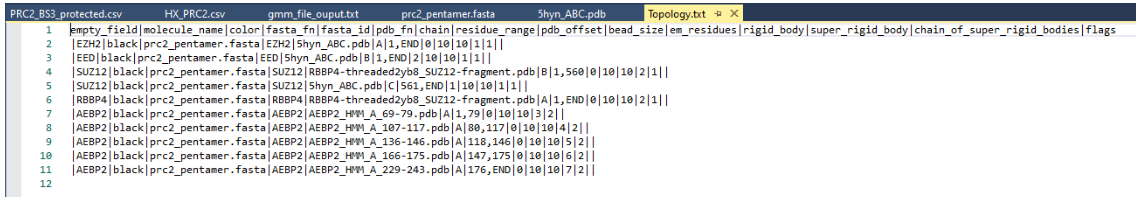

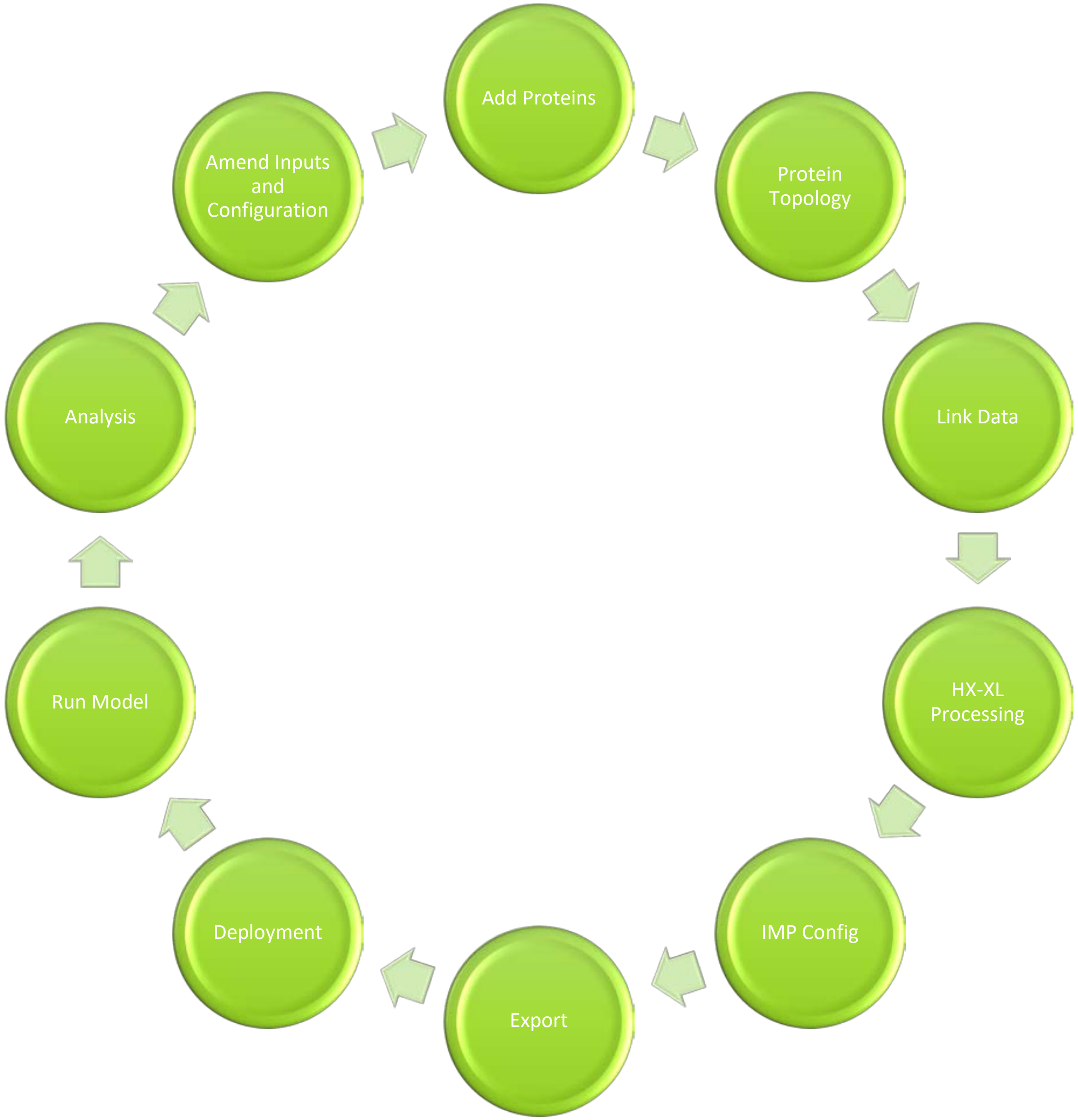

IMProv lifecycle

- Prepare Export bundle using MassSpecStudio wizard steps
- Prepare Deployment
- Run Modeling job
- Review

Example 2: IMProv PRC2

- Present another IMProv project

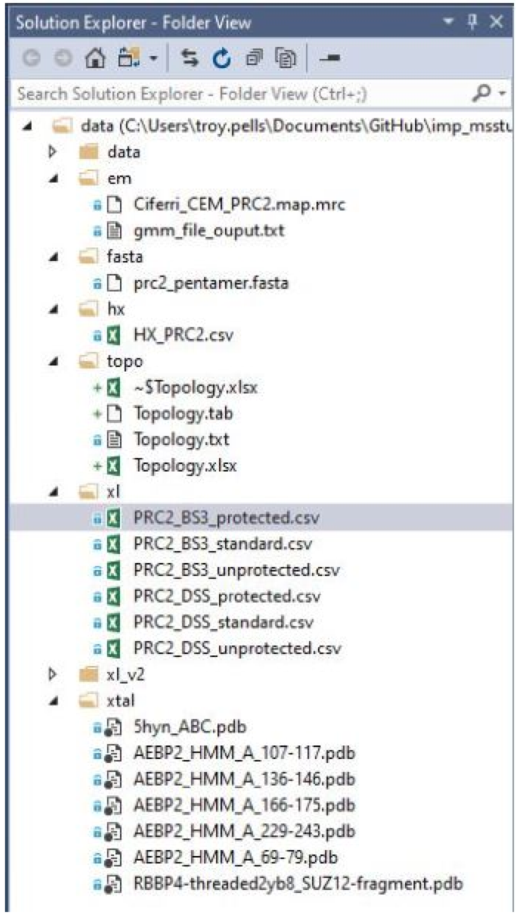

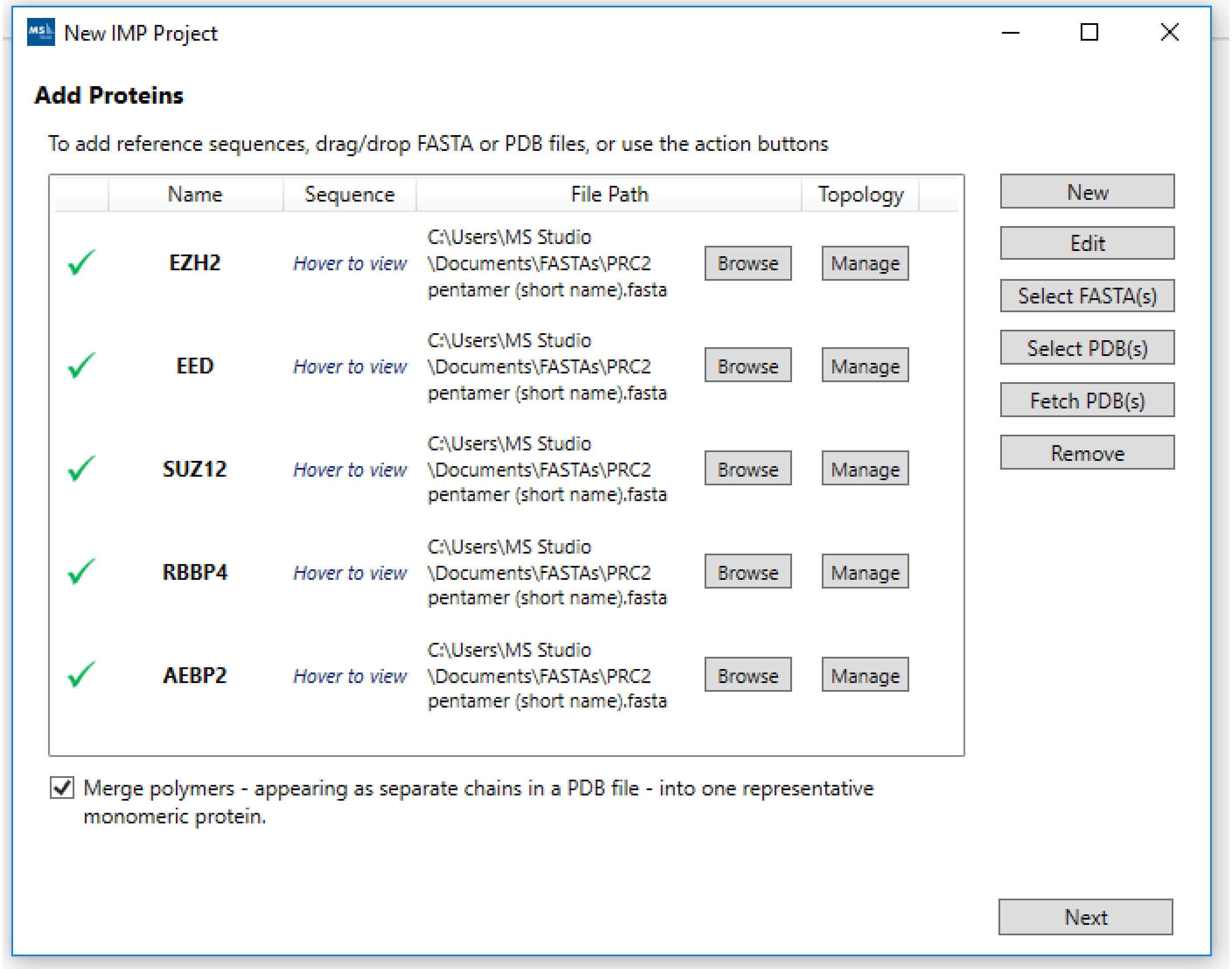

Wizard Steps

- **Add Proteins.**
- Add Protein Topology
- Add Link Data
- HX-XL Classification
- Configure IMP

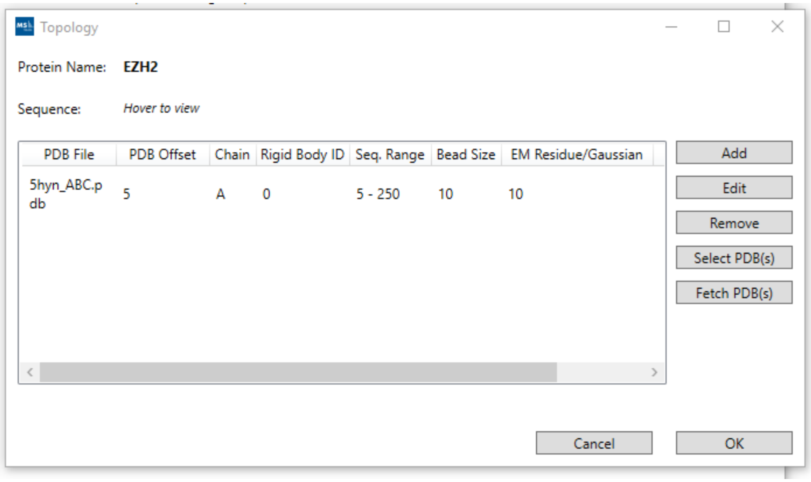

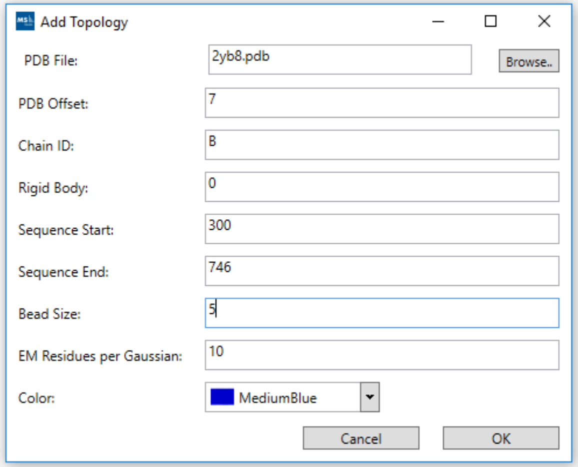

Wizard Steps

- Add Proteins.
- Add Protein Topology
- Add Link Data
- HX-XL Classification
- Configure IMP

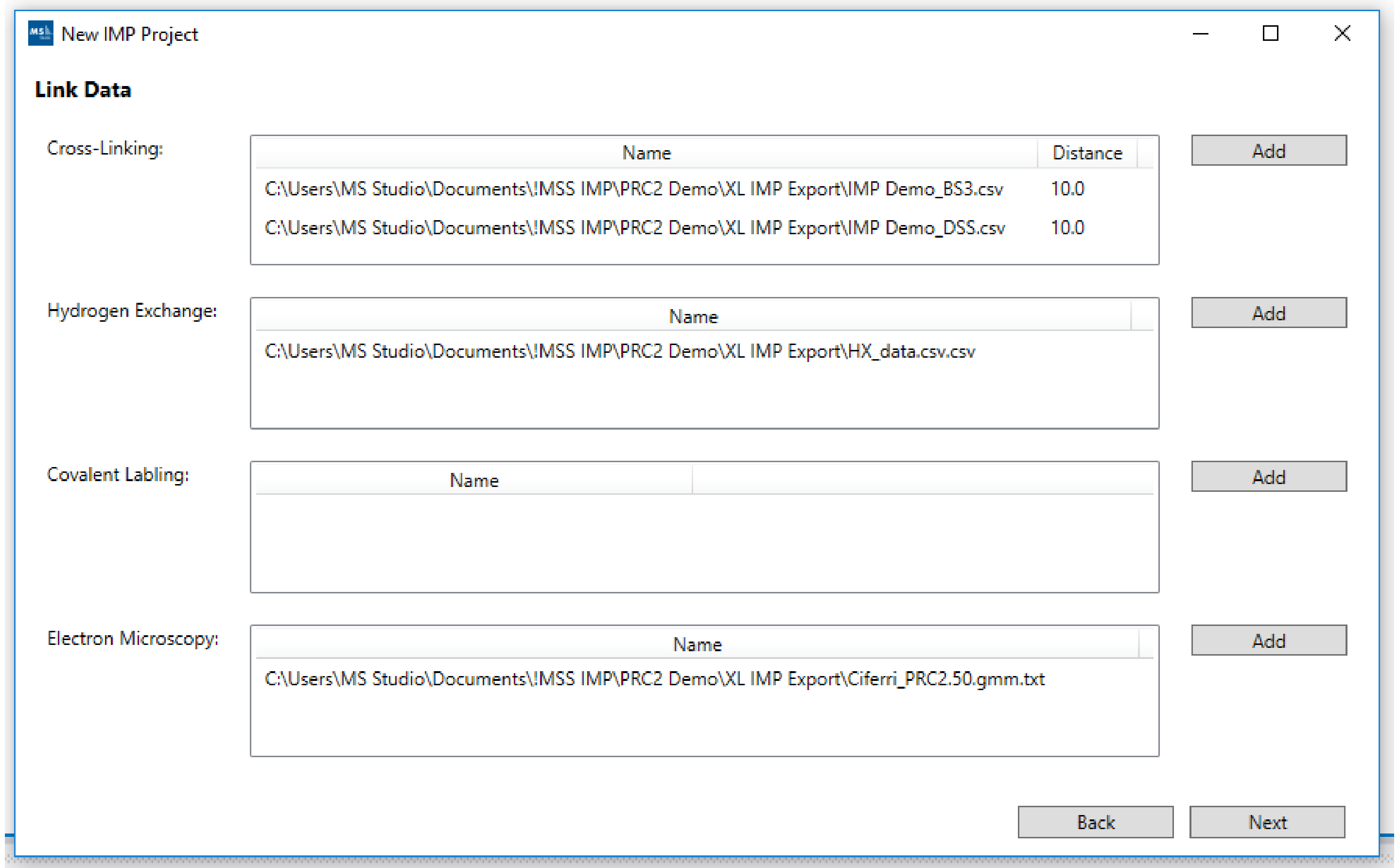

Wizard Steps

- Add Proteins.
- Add Protein Topology
- **Add Link Data**
- HX-XL Classification
- Configure IMP

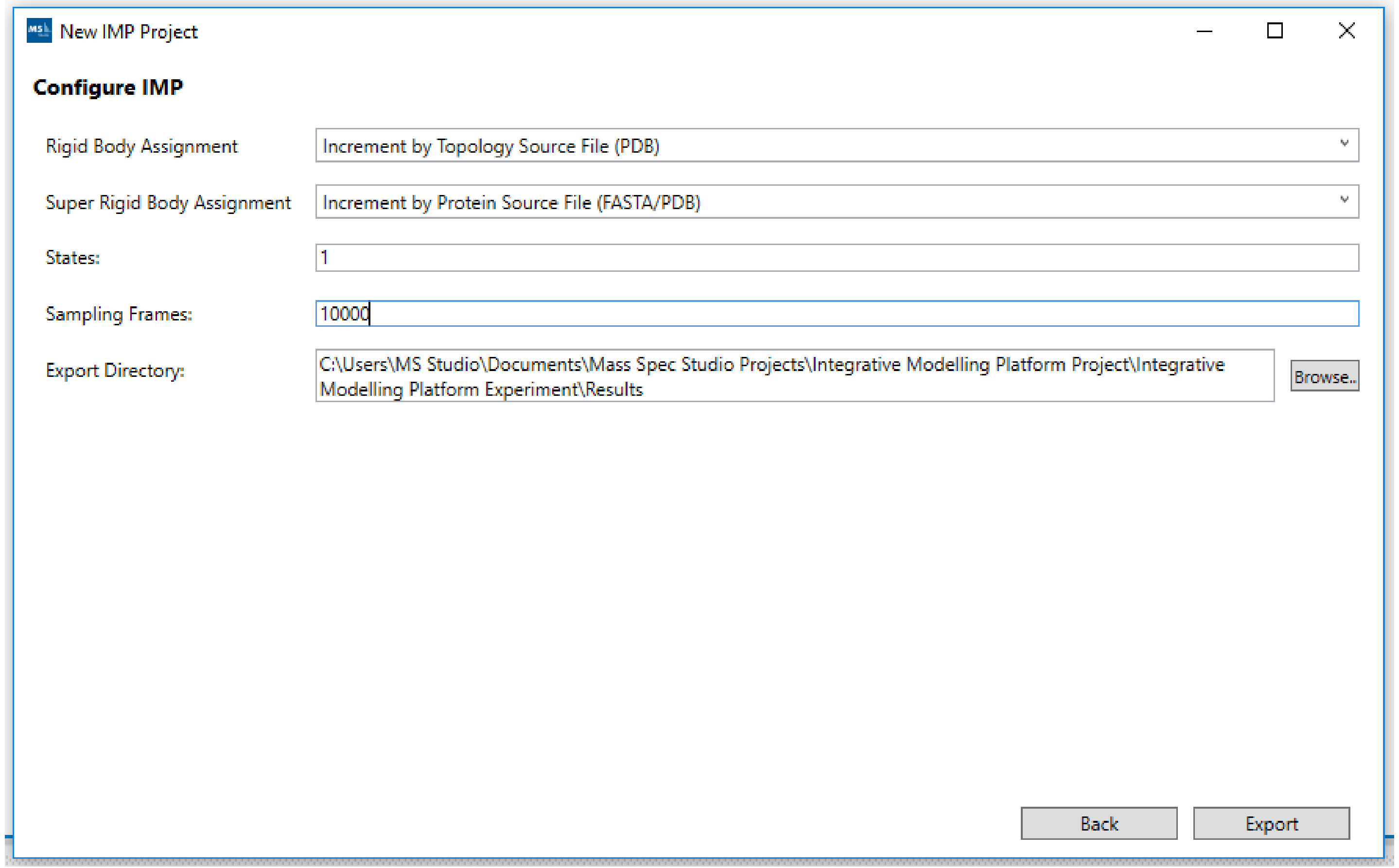

Wizard Steps

- Add Proteins.
- Add Protein Topology
- Add Link Data
- HX-XL Classification
- **Configure IMP**

Final remarks on IMProv

- We have seen two IMProv projects.

Deployment of the Project

- Deployment follows a 3 step setup process.
- Deployment to Cedar.
- Deployment to AWS.

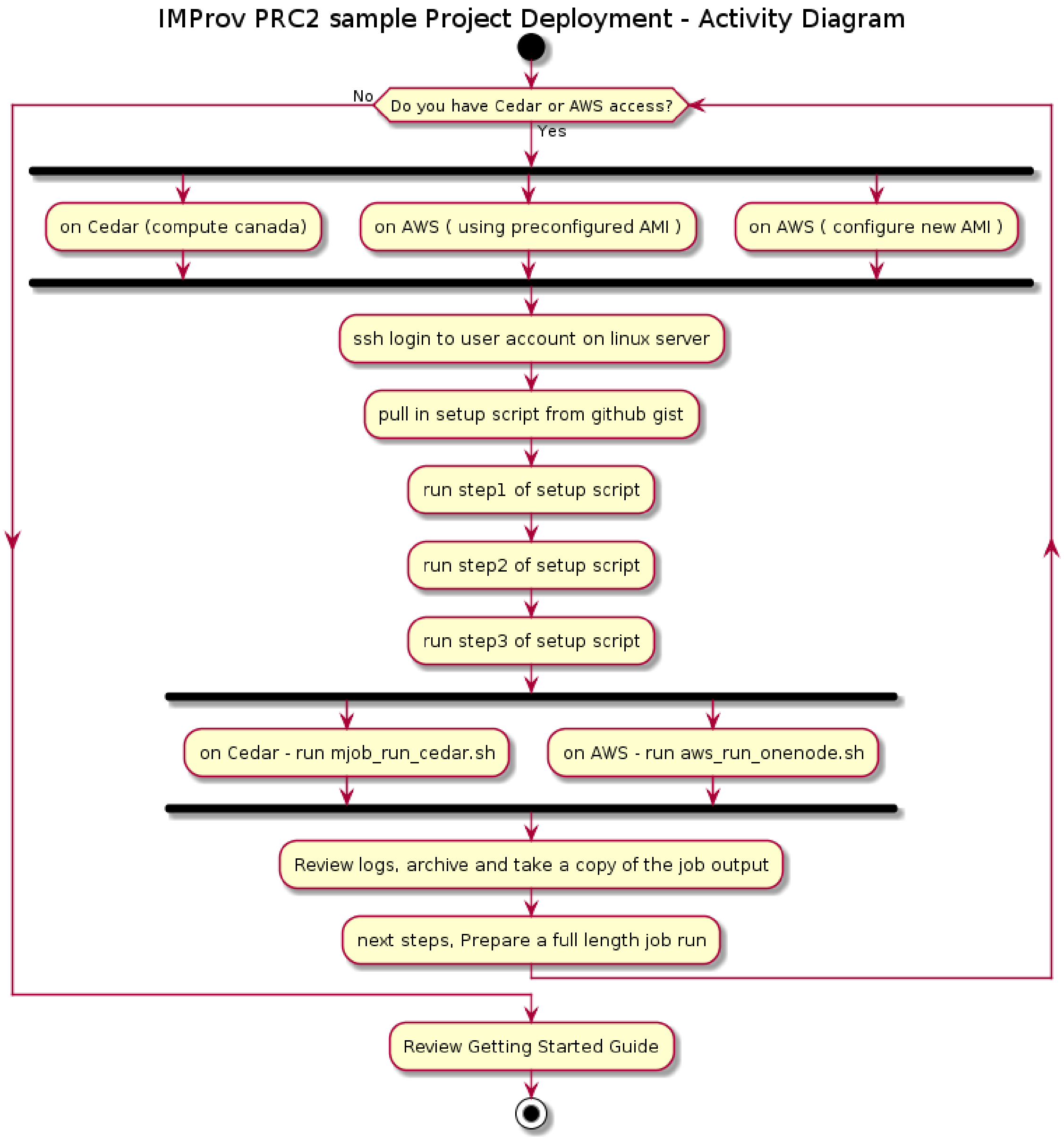

Deployment steps

- Launch the modeling run on a multi-cpu machine
- MPI is the message passing interface used to leverage multi-cpu machine when performing the Monte-Carlo sampling.
- The step1 through step3 scripts mentioned are provided in the sample PRC2 project on github. The link is given in the reference section at the end.

Deployment: Cedar

- The github repo for imp_msstudio_init contains the online tutorials together with the PRC2 sample project. The bootstrap scripts are also available.
- https://github.com/pellst/imp_msstudio_init

- **IMProv_on_Cedar_tut.md**: https://github.com/pellst/imp_msstudio_init/blob/master/IMProv_on_Cedar_tut.md
- #### get the setup script from github gist and review before running:
- ~~~
- curl -LOk https://gist.githubusercontent.com/pellst/4853822ea5ca74785af61d0ad39cf84d/raw/uoc_mss_prep_step1.sh
- chmod 755 uoc_mss_prep_step1.sh
- ~~~

- #### run the script uoc_mss_prep_step1.sh in order to get the sample folders and scripts setup
- ~~~
- ./uoc_mss_prep_step1.sh
- ~~~

- #### in the folder /scratch/$USER/imp/imp_msstudio_init-master/mss_out/imp_model, the following shell scripts are now available
- ~~~
- uoc_mss_prep_step1.sh
- uoc_mss_prep_step2.sh
- uoc_mss_prep_step3.sh
- ~~~

Deployment: AWS

- The github repo for imp_msstudio_init contains the online tutorials together with the PRC2 sample project. The bootstrap scripts are also available.
- https://github.com/pellst/imp_msstudio_init

- **IMProv_on_AWS_tut.md**: https://github.com/pellst/imp_msstudio_init/blob/master/IMProv_on_AWS_tut.md
- # make use of this gist to get the prep_step* shell scripts located here
- # /shared/imp/imp_msstudio_init-master/mss_out/imp_model
- curl -LOk https://gist.githubusercontent.com/pellst/9f7ad519133dae87f8f813b506b45aac/raw/**aws_mss_prep_step1.sh**
- chmod 755 aws_mss_prep_step1.sh
- ./aws_mss_prep_step1.sh

- # prepare anaconda install
- #/shared/imp/imp_msstudio_init-master/mss_out/imp_model/aws_mss_prep_step2.sh
- #/shared/imp/imp_msstudio_init-master/mss_out/imp_model/aws_mss_prep_step3.sh

- A new folder is created with the run number (imp_model_nn) as a copy of the imp_model folder content.
- The data folder is referenced and is not duplicated when performing multiple run’s in parallel on a HPC platform (replicate run’s). Avoid confusion with MPI which will be performed within a single run and hence each instance thereof in a parallel run of for example 3 modeling jobs at the same time (ie: 3 replicates each performing independent MC sampling).
- A subfolder of imp_model_nn is the output folder which has sub-folders
  - pdbs
  - rmfs
- When run on 16 cpu. There will be one .rmf3 file per cpu (0 through 15)
- stat.*.out and stat_replica.*.out files

Modeling Run Review

Note that the modeling run generates several files in the imp_model_nn folder (where nn is the run number given)

- included.*.db
- excluded.*.db
- missing.*.db

The trace files are created as

- prep_hyperp_imp_v2.log
- slurm-nnnnnnn.out

Deployment: Wrap-up

- Deployment can be to your local PC, AWS or on Compute Canada HPC platform (Cedar) .
- Performing a test run with the sample PRC2 project enables one to validate the installation and confirm that IMP together with Anaconda are correctly installed.
- Your own IMProv project can then be copied to the deployment folder and launch a modeling run. Start with 100 frames for testing purposes.
- Check the output folder and log files to confirm that MPI is using the cpu count you specified.

Summary

- IMProv lifecyle.
- Online guides and tutorials.
  - IMProv_msstudio_tut.md (tiny url)
  - uml_activity_diag_improv.svg
  - IMProv_on_AWS_tut.md
  - IMProv_on_Cedar_tut.md

Assessment and Evaluation

- How is the python driver script and IMPConfig.yaml a convenience?
  - what would you need to do if there are not available?
- IMProv comprises file preparation and deployment. How would you go about performing each?

- Did you find this guided tutorial helpful?

• Abbreviations

- **Cryo-EM:** cryoelectron microscopy | https://www.sciencedirect.com/science/article/pii/S0304416517302374
- **FDR**: False Discovery Rate | https://www.bioinfor.com/fdr-tutorial/
- **HPC**: High Performance Computing | https://docs.computecanada.ca/wiki/Getting_started
- **HX-MS**: Hydrogen eXchange Mass Spectrometry | https://neu.hxms.com/research/tutorial_theory.htm#:~:text=Hydrogen%20exchange%20(HX)%20combined%20with,of%20proteins%20and%20protein%20structure.
- **IMP**: Integrative Modeling Platform | https://integrativemodeling.org/
- **PMI**: Python Modeling Interface | https://integrativemodeling.org/
- **PRC2**: Polycomb Repressive Complex 2 | https://www.ncbi.nlm.nih.gov/pmc/articles/PMC5008062/
- **SLURM**: Simple Linux Utility for Resource Management | https://en.wikipedia.org/wiki/Slurm_Workload_Manager
- **XL-MS**: Crosslinking Mass Spectrometry | https://www.technologynetworks.com/proteomics/articles/cross-linking-mass-spectrometry-a-key-player-in-the-structural-biologists-toolbox-322446
- **FASTA**: The FASTA format is sometimes also referred to as the "Pearson" format (after the author of the FASTA program and ditto format). | https://www.bioinformatics.nl/tools/crab_fasta.html ; https://en.wikipedia.org/wiki/FASTA_format
- **PDB**: The Protein Data Bank (pdb) file format is a textual file format describing the three-dimensional structures of molecules held in the Protein Data Bank | https://pdb101.rcsb.org/learn/guide-to-understanding-pdb-data/introduction ; https://www.rcsb.org/
- **AWS**: Amazon Web Services | https://aws.amazon.com/console/
- **Cedar**: Compute Canada HPC Cluster | https://status.computecanada.ca/
- **Linux**: Operating System, RedHat Enterprise Linux (or variants, such as CentOS or Scientific Linux) |
- **MC**: Monte Carlo Sampling

• References

- **IMProv_msstudio_tut.md:** https://github.com/pellst/imp_msstudio_init/blob/master/IMProv_msstudio_tut.md
- **uml_activity_diag_improv.svg**: https://raw.githubusercontent.com/pellst/imp_msstudio_init/master/uml_activity_diag_improv.svg
- **IMProv_uml_diag.png**: https://github.com/pellst/imp_msstudio_init/blob/master/IMProv_uml_diag.png
- **IMProv_on_Cedar_tut.md**: https://github.com/pellst/imp_msstudio_init/blob/master/IMProv_on_Cedar_tut.md
- **IMProv_on_AWS_tut.md**: https://github.com/pellst/imp_msstudio_init/blob/master/IMProv_on_AWS_tut.md
